# Adaptive mechanisms that provide competitive advantages to marine bacteroidetes during microalgal blooms

**DOI:** 10.1038/s41396-018-0243-5

**Published:** 2018-07-30

**Authors:** Frank Unfried, Stefan Becker, Craig S. Robb, Jan-Hendrik Hehemann, Stephanie Markert, Stefan E. Heiden, Tjorven Hinzke, Dörte Becher, Greta Reintjes, Karen Krüger, Burak Avcı, Lennart Kappelmann, Richard L. Hahnke, Tanja Fischer, Jens Harder, Hanno Teeling, Bernhard Fuchs, Tristan Barbeyron, Rudolf I. Amann, Thomas Schweder

**Affiliations:** 1 Pharmaceutical Biotechnology, University Greifswald, Greifswald, Germany; 2 Max Planck Institute for Marine Microbiology, Bremen, Germany; 3 Institute of Marine Biotechnology, Greifswald, Germany; 4 MARUM, Center for Marine Environmental Sciences at the University of Bremen, Bremen, Germany; 5 Institute for Microbiology, University Greifswald, Greifswald, Germany; 6 DSMZ, Braunschweig, Germany; 7 National Center of Scientific Research/Pierre and Marie Curie University, Paris, France; 8 UMR 7139 Marine Plants and Biomolecules, Station Biologique de Roscoff, Roscoff, Bretagne France

## Abstract

Polysaccharide degradation by heterotrophic microbes is a key process within Earth’s carbon cycle. Here, we use environmental proteomics and metagenomics in combination with cultivation experiments and biochemical characterizations to investigate the molecular details of in situ polysaccharide degradation mechanisms during microalgal blooms. For this, we use laminarin as a model polysaccharide. Laminarin is a ubiquitous marine storage polymer of marine microalgae and is particularly abundant during phytoplankton blooms. In this study, we show that highly specialized bacterial strains of the Bacteroidetes phylum repeatedly reached high abundances during North Sea algal blooms and dominated laminarin turnover. These genomically streamlined bacteria of the genus *Formosa* have an expanded set of laminarin hydrolases and transporters that belonged to the most abundant proteins in the environmental samples. In vitro experiments with cultured isolates allowed us to determine the functions of in situ expressed key enzymes and to confirm their role in laminarin utilization. It is shown that laminarin consumption of *Formosa* spp. is paralleled by enhanced uptake of diatom-derived peptides. This study reveals that genome reduction, enzyme fusions, transporters, and enzyme expansion as well as a tight coupling of carbon and nitrogen metabolism provide the tools, which make *Formosa* spp. so competitive during microalgal blooms.

## Introduction

Phytoplankton blooms produce large quantities of beta-glucans, such as laminarin, a soluble β-1,3-glucan with β-1,6 side chains. The breakdown of these polysaccharides by heterotrophic microbes is a central part of the marine carbon cycle. Diatoms alone are estimated to produce ~ 5–15 Gt of laminarin per year as their storage compound, making it a major food resource for heterotrophic marine organisms [1]. Bacterial laminarinase activities are abundant in ocean surface waters, but also within deeper parts of the water column and in sediments [2, 3]. This suggests laminarin-degrading bacteria and their laminarinases are common across the oceans. How bacteria compete for this abundant labile energy substrate is therefore of relevance for a better understanding of the marine carbon cycle. Although partially studied with model organisms in the laboratory [4–7], the enzymes used for laminarin degradation by microbes in the wild remain largely unknown or uncharacterized.

For complete degradation of one polysaccharide, microbes must have an adapted glycolytic pathway that contains multiple enzymes, which individually address each of the different glycosidic linkages and structural compositions present in the macromolecule. The genes of glycan-degrading pathways cluster in operons named polysaccharide utilization loci (PULs). Recent works suggest that each polysaccharide requires a corresponding PUL (for review, see Grondin et al. [8]). Horizontal gene transfer, vertical inheritance, and gene loss distribute PULs asymmetrically among genomes of microbes, creating the molecular basis for polysaccharide resource partitioning [9–11]. This might explain the occurrence of diverse bacterial communities in the human gut [12–15] or in the oceans, whose members rely on different degradation products of the same polysaccharide to co-exist [16–19]. However, it remains unclear whether the degradation of complex carbohydrates is a community effort or mainly driven by highly specialized individual strains. Furthermore, how microbes effectively compete for the same polysaccharide resource, such as the abundant laminarin, is currently unknown.

In previous studies, we reported the high abundance (up to 24% of all bacteria) of the flavobacterial genus *Formosa* during diatom-dominated spring blooms off the North Sea island Helgoland [17, 18]. Furthermore, high laminarin concentrations were measured at the same sampling site [20]. Together, these findings suggested that *Formosa* spp. are prominent candidates for the recycling of laminarin during spring microalgae blooms.

In this study, we explored molecular strategies, which provide competitive advantages to the genus *Formosa* during microalgal blooms in general and for laminarin utilization in particular. We examined two strains, *Formosa* Hel3_A1_48 (referred to as strain A) and *Formosa* Hel1_33_131 (strain B), both of which were isolated from the same sampling location [21], and which are representative of two distinct taxonomical clades found during phytoplankton blooms [22]. The combination of high-resolution metaproteomics and metagenomics of spring bloom water samples with the detailed proteomic and biochemical characterization of the respective PUL in a cultured model strain (*Formosa* B) allowed us to show that a specialized enzyme repertoire represents one of the adaptive mechanisms that provide a competitive advantage in substrate exploitation. Using laminarin as a model substrate, we demonstrate how a microalgal glycan resource can promote the enrichment of individual dominating taxa from an initially diverse microbial community with similar metabolic functions. Our data indicate that *Formosa* B tightly couples glycan utilization with the uptake of nitrogen compounds. This suggests that a balanced carbon and nitrogen diet is required for competitive laminarin utilization during phytoplankton blooms.

## Materials and methods

### Growth experiments and physiological characterization

The investigated strains *Formosa* sp. Hel1_33_131 (*Formosa* strain B) and *Formosa* sp. Hel3_A1_48 (*Formosa* strain A) were isolated by dilution cultivation during a spring and a summer phytoplankton bloom, respectively, from surface water near the North Sea island Helgoland in the German Bight [21]. Growth experiments were performed in a modified HaHa medium [21] (with 0.1 g L^−1^ peptone, 0.1 g L^−1^ casamino acids, 0.1 g L^−1^ yeast extract, 200 μM NH_4_Cl, and 16 μM KH_2_PO_4_) with defined carbon sources as substrates at 12 °C during gentle shaking at 55 rpm. For the proteome analyses, described below, d-glucose and laminarin (L9634, Sigma-Aldrich Chemie GmbH, Taufkirchen, Germany) were used as carbon sources (concentrations: 2 g L^−1^). In addition, the utilization of chitin (SAFSC9213, VWR) was tested in this medium and these cultures were used as a control condition for the in vitro proteome analyses with glucose and laminarin. All growth experiments were carried out in triplicates. Cells were harvested by centrifugation (15 min; 9500 × *g*; 4 °C), and the resulting pellets and supernatants were stored at − 80 °C until use.

### Genome sequencing, assembly, and annotation

For genome sequencing of the strains *Formosa* A (Hel3_A1_48) and B (Hel1_33_131) DNA was extracted according to the protocol of Zhou et al. [23]. Sequencing was performed at LGC Genomics (Berlin, Germany) using the 454 GS FLX Ti platform (454 Life Sciences, Branfort, CT, USA) using standard shotgun libraries. Draft genomes were assembled with Newbler v2.6 for Hel3_A1_48 from 640,093 reads (406,983,286 bp) and for Hel1_33_131 from 636,323 reads (410,253,204 bp), yielding 2,025,184 bp (77 contigs) and 2,727,763 bp (61 contigs), respectively. The remaining gaps were closed by PCR and Sanger sequencing, yielding circular assemblies of 2,016,454 bp for Hel3_A1_48 (*Formosa* A) and 2,735,158 bp for Hel1_33_131 (*Formosa* B). Gene prediction and annotation (including the phylogeny-guided carbohydrate-active enzyme (CAZyme) annotations provided in Supplementary Table S2) were performed as described previously [24]. Further bioinformatic analyses are described in Supplementary Information. Annotated genome sequences were submitted to NCBI’s GenBank with the accession numbers CP017259.1 for *Formosa* sp. Hel3_A1_48 (*Formosa* strain A) and CP017260.1 for *Formosa* sp. Hel1_33_131 (*Formosa* strain B).

### Proteome analyses

The soluble intracellular proteome, the enriched membrane-associated proteome, and the soluble extracellular proteome was characterized from exponentially growing cells of *Formosa* strain B. Details of the protein extraction and subproteome enrichment can be found in Supplementary Information.

Peptides were subjected to a reversed phase C18 column chromatography on a nano ACQUITY-UPLC (Waters Corporation, Milford, MA, USA) and separated as described by Otto et al. [25]. Mass spectrometry (MS) and MS/MS data were recorded using an online-coupled LTQ-Orbitrap Classic mass spectrometer (Thermo Fisher Scientific Inc., Waltham, MA, USA). We searched MS spectra against a target-decoy protein sequence database including sequences of *Formosa* B (Hel1_33_131) and of common laboratory contaminants.

Protein searches were performed using MaxQuant with the integrated Andromeda engine [26] with a peptide level FDR (false discovery rate) set to 0.01 (1%). Only proteins that could be detected in at least two out of three replicates were counted as identified. The automatically calculated iBAQ values (intensity-based absolute quantification; i.e., peak area divided by the sum of all theoretical peptides) were used to manually calculate riBAQ values (relative iBAQ; giving the relative protein abundance in % of all proteins in the same sample, [27]) for semiquantitative comparisons between samples from different nutrient conditions. Tests for differential expression were performed using Perseus [28] v. 1.6.1.1 with Welchs *t* test (permutation-based FDR 0.05).

The mass spectrometry proteomics data are available through the ProteomeXchange Consortium (http://proteomecentral.proteomexchange.org) via the PRIDE partner repository [29] with the dataset identifier PXD007934.

### Biochemical enzyme characterizations

The cloning of the FbGH17A gene (locus tag FORMB_24720) and the FaGH17B gene (locus tag FORMB_24740) is described in Supplementary Information. Cloning of the FbGH30 gene is described by Becker et al. [20]. Detailed information on the overexpression, enzyme refolding, and purification of these proteins can be found in Supplementary Information. For enzyme characterizations laminarin from *Laminaria digitata* (0.1% [w/v]; Sigma) was hydrolyzed over the course of 60 min at 37 °C with 100 nM purified enzyme (~ 5 µg mL^−1^ of FbGH30, FbGH17A, or FbGH17B) in 50 mM MOPS buffer at pH 7. The preparation and purification of debranched laminarin as well as the determination of kinetic parameters of the three enzymes FbGH30, FbGH17A, and FbGH17B acting on native and debranched laminarin is explained in Supplementary Information. High performance anion exchange chromatography with pulsed amperometric detection (HPAEC-PAD) was applied for qualitative product analysis of the enzyme reactions (see Supplementary Information).

### Protein crystallization and structure solution

Crystals of FbGH17A were obtained by hanging drop vapor diffusion of the protein with 12.3 mg mL^−1^ mixed 1:1 with a “well solution” (0.03 M MgCl_2_, 0.1 M MOPS (pH 7), 9% PEG8K) supplemented with 15% ethylene glycol. The crystals were cryoprotected prior to freezing in the “well solution” supplemented with ethylene glycol to a final concentration of 30%. Crystals were frozen by flash freezing in liquid nitrogen in nylon loops. X-ray diffraction data were collected at the DESY P11 beamline. The structure was solved by molecular replacement using PHASER in the phenix suite [30, 31] using the pdb 4wtp [32]. The model was built using BUCCANEER [33] and Coot, refined in REFMAC5 [34], and validated and deposited with pdb code 6FCG.

## Results

### Genome properties and phylogeny

We sequenced and annotated the genomes of the *Formosa* strains A and B. Both have a single chromosome with a GC content of 36.4 and 36.6%, respectively. With 2,016,454 bp (strain A) and 2,735,158 bp (strain B) they possess small genomes compared with other marine polysaccharide-degrading *Flavobacteriia* [4, 24, 35, 36]. Strain A has 1913 predicted genes including 1866 coding sequences (CDS), 40 tRNAs genes, and 2 rRNA operons (identical 5 S, 16 S, 23 S rRNA genes), whereas the strain B genome encodes 2675 predicted genes with 2628 CDS, 39 tRNAs genes, and 2 rRNA operons (identical 5 S, 16 S, 23 S rRNA genes).

Phylogenetic analyses based on 16 S rRNA gene sequences indicate that the *Formosa* strains A and B are representatives of two previously uncultured clades of the genus *Formosa*. These occur not only in the North Sea, but also in surface waters from coastal and open ocean sites throughout the world (Supplementary Figure S1). Of the 33 full-length *Formosa* 16 S rRNA sequences obtained from 2009 spring bloom bacterioplankton [17], 16 were > 99% identical to *Formosa* sp. Hel1_33_131 (*Formosa* B) (Supplementary Figure S1).

### Formosa genomes encode PULs for laminarin degradation

Genome annotation suggested that the *Formosa* strains A and B are specialized polysaccharide degraders, which concentrate their genetic potential on a small set of sugars. *Formosa* A contains seven PULs (Supplementary Figure S2) and 28 glycoside hydrolases (Supplementary Table S1), whereas *Formosa* B contains six PULs (Supplementary Figure S3) and 21 glycoside hydrolases (Supplementary Table S1). This is a very small repertoire, even compared with other marine *Bacteroidetes* isolated from algal blooms [7]. These small CAZyme repertoires contrast particularly with those of generalist polysaccharide degraders isolated from macroalgae, such as *Formosa agariphila* [24], which have broad polysaccharide-degrading capacity. *F*. *agariphila* has, for example, a genome size of 4.48 Mbp, and 84 glycoside hydrolases in 13 PULs (Supplementary Table S1).

To functionally characterize laminarin-specific PULs of the *Formosa* strains A and B, we searched the genomes for enzymes belonging to known laminarinase-containing families (Supplementary Table S1). We found putative laminarinases of the families GH16 and GH17 but also enzymes of the GH3 and GH30 families as well as a member of the newly described GH149 family [37], located in close proximity to TonB-dependent receptors (TBDR) and SusD-like proteins, which are indicators of PULs [38, 39]. Our results suggest that there are three putative laminarin-specific genomic PULs in both *Formosa* strains (Supplementary Figure S2–S5).

The laminarin PULs 1 and 2 of *Formosa* A and B revealed a high synteny with PULs from other bacteroidetal strains (Supplementary Figure S4) from North Sea surface water [21, 36, 40]. This points to a potential for competition between those groups, but also suggests that this part of the laminarin utilization machinery is highly conserved. However, the *Formosa* B PULs 1 and 2 are enlarged with laminarinases and transporters that are partially not present in the other bacteria. Moreover, the entire PUL 3 of *Formosa* B is missing in these other strains (Supplementary Figure S5). Instead, *Formosa* B’s PUL 3 shows synteny to PULs of other marine *Flavobacteriia*, which do, however, not possess the PULs 1 and 2 (Supplementary Figure S5).

### Laminarin elicits the expression of specific polysaccharide utilization loci in Formosa

**Fig. 1:**
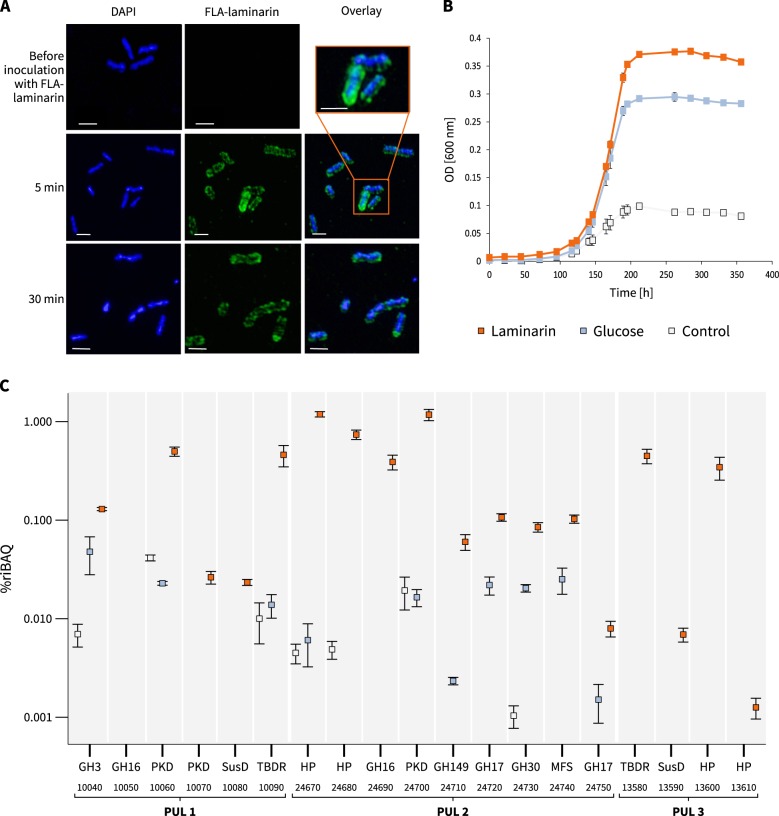
Laminarin utilization of *Formosa* B. **a** SR-SIM of *Formosa* B cells before inoculation with FLA-laminarin at 5 and 30 min after incubation with FLA-laminarin. Images show cell staining by DAPI (left, blue), FLA-laminarin (middle, green), and an overlay showing both FLA-laminarin staining and DAPI (right). Scale bar = 1 µm **b** Growth curves of three biological replicates at 12 °C in modified HaHa_100V medium [21] with 2 g L^−1^ laminarin or 2 g L^−1^ glucose. The “control” culture contained only 0.1 g L^−1^ peptone, 0.1 g L^−1^ yeast extract, and 0.1 g L^−1^ casamino acids but no additional carbon sources. **c** Expression profile and gene organization of the laminarin utilization PULs 1–3 in *Formosa* B. Relative protein abundances (in %riBAQ) of PUL-encoded proteins detected in the membrane protein fractions of each three independent cultures grown on laminarin (orange), glucose (blue), and chitin (control, gray) are shown (for riBAQ values see Supplementary Tables S2A and S3). Putative protein functions (e.g., GH3) and the respective locus tags (e.g., 10040) are indicated. The squares represent the mean values of the replicates for every protein and each substrate. The error bars refer to the standard error of the mean. Proteins that could be detected in at least two out of three independent biological replicates of each substrate condition are shown (for individual replicate numbers see Supplementary Table S2A). GH, glycoside hydrolase; PKD, PKD-domain containing protein; SusD, SusD-family protein; HP, hypothetical proteins; MFS, major facilitator superfamily; TBDR, TonB-dependent receptor

Incubation experiments with fluorescently labeled (FLA) laminarin revealed the ability of *Formosa* B to quickly react and take up laminarin. *Formosa* B accumulated high amounts of FLA-laminarin after just 5 min of incubation (Fig. 1a). Additionally, the halo-like staining pattern showed that the FLA-laminarin was imported into the periplasm of the cells by a “selfish” uptake mechanism [41, 42]. Selfish substrate uptake is dependent on the presence of SusCD-like transporters and secures an enrichment of substrate in the periplasmic space without diffusive loss [42].

To elucidate the metabolism of *Formosa* B on laminarin and to verify whether laminarin specifically controls the expression of the genomically detectable PULs we performed cultivation experiments with this bacterium with purified laminarin as growth substrate. Growth curves of *Formosa* B in HaHa medium with laminarin, glucose and only protein extracts, respectively, are shown in Fig. 1b. We used proteomics to record the global protein expression patterns with these substrates. We investigated (i) the soluble intracellular proteome, (ii) the enriched membrane proteome, and (iii) the extracellular proteome (see Supplementary Information and Supplementary Tables S2A–C). These comparative analyses showed that although glucose is the monomer of laminarin, the utilization of either carbon source led to quite different proteomic signatures in different functional protein categories, such as in nucleotide, lipid, and coenzyme metabolism as well as in carbohydrate metabolism and transport (Supplementary Figure S6). About 100 proteins were significantly higher abundant or only found in laminarin incubations in *Formosa* B (Supplementary Figure S7, Supplementary Table S3). Of all three substrates, laminarin elicited the strongest expression of the three laminarin PULs of *Formosa* B (Fig. 1c), which is indicative of specific and tightly controlled expression. The SusD-like protein (FORMB_10080) of PUL 1 and the GH16 (FORMB_m24690) of PUL 2 were exclusively expressed with laminarin but not with the other substrates. Furthermore, the expression of PUL 3 was exclusively induced by laminarin and not detectable with glucose or only peptone (Fig. 1c). The specific response of the *Formosa* PULs to laminarin and not to glucose implies that the three-dimensional structure of laminarin might be the key to induce the expression of these PULs.

### Biochemical analysis of laminarinases expressed by Formosa spp

**Fig. 2:**
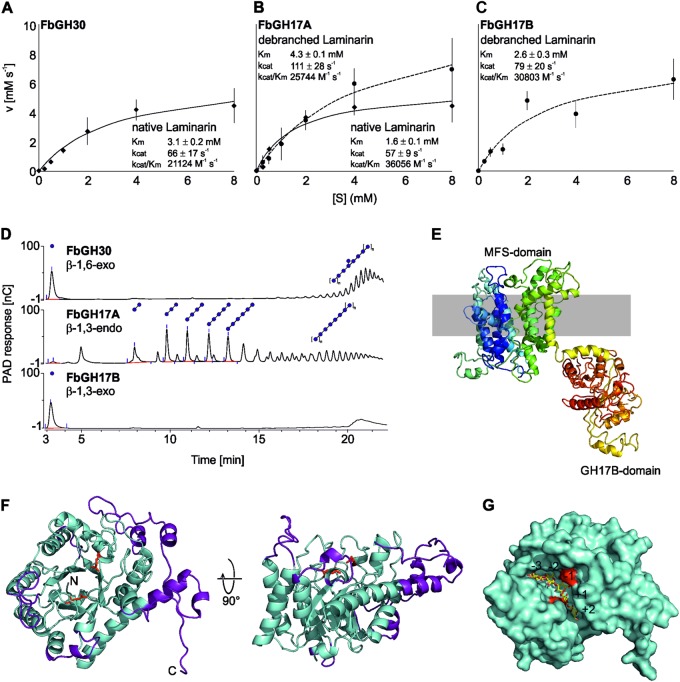
Biochemical characterization of different laminarinases from *Formosa* B. Michaelis–Menten Kinetic (**a**) of FbGH30 on native laminarin, **b** of FbGH17A on native and debranched laminarin and **c** of FbGH17B on debranched laminarin (native laminarin is illustrated by the solid lines and debranched laminarin by the dashed lines). **d** Visualization of all three enzymatic activities was done using HPAEC-PAD. FbGH30 hydrolyzed native laminarin. After this debranching reaction, the laminarin was purified to remove glucose for the following steps. This debranched laminarin was used in the FbGH17A reaction. FbGH17B hydrolyzed the products of the previous FbGH17A reaction without any further purification in between. **e** 3D structure model of both the MFS-domain and the associated FbGH17B-domain and its potential arrangement within the inner-membrane. The modeling was performed using Phyre2. **f** The overall structure of FbGH17A is displayed in cyan with the additions colored purple. Highlighted in red are the catalytic residues. The N- and C-termini are labeled. **g** A surface view of FbGH17A with a modeled substrate complex shown as sticks in yellow from a GH17 transferase of *Rhizomucor miehei* with laminaritriose and laminaribiose in the −3 to −1 and + 1 to + 2 subsites, respectively

To functionally characterize PUL-encoded proteins and to map the laminarin degradation pathway, we cloned and biochemically analyzed putative laminarinases the function of which could not be merrily solved by comparative sequence analyses with known enzyme functions. We cloned and examined the genes encoding FbGH17A (locus tag: FORMB_24720), FaGH17B (locus tag: FORMB_24740), and FbGH30 (locus tag: FORMB_24730). As all three proteins are encoded in a single gene cluster, we hypothesized that these enzymes might work together in spatial proximity. To test this hypothesis, we conducted a series of biochemical experiments, which revealed that the FbGH30 enzyme hydrolyzed the β-1,6-linked glucose side chains of laminarin (*K*_M_: 3.1 ± 0.2 mM and K_cat_/K_M_: 21124 M^−1^s^−1^) (Fig. 2a), whereas it was inactive on the debranched substrate. The enzyme FbGH17A hydrolyzed both the debranched laminarin product of FbGH30 and the native laminarin, although with a markedly higher specific activity on the debranched product (K_M_: 1.6 ± 0.1 mM; K_cat_/K_M_: 36056 M^−1^s^−1^) than on laminarin itself (K_M_: 4.3 ± 0.1 mM; K_cat_/K_M_: 25744 M^−1^s^−1^) (Fig. 2b). Preference for debranched laminarin was even more pronounced with FbGH17B, which only hydrolyzed debranched laminarin (K_M_: 2.6 ± 0.3 mM; K_cat_/K_M_: 30803 M^−1^s^−1^) (Fig. 2c) and was inactive on the branched form.

To elucidate how these enzymes work together in successive laminarin degradation, we used high-performance liquid chromatography method with photo diode array detection analyses. The data indicated an enzymatic functional cascade in three steps (Fig. 2d): The exo-acting β-1, 6-glucosidase FbGH30 removes the glucose side chains from laminarin (Supplementary Figure S8A). The endo-acting β-1, 3-glucan hydrolase FbGH17A degrades the remaining debranched laminarin into oligosaccharides (Supplementary Figure S8B). The exo-acting β-1, 3-glucosidase FbGH17b processes these oligosaccharides into glucose (Supplementary Figure S8C). FbGH17b is part of a multi-modular protein, which is encoded by a gene that also codes for an N-terminal major facilitator superfamily (MFS) transporter, suggesting that hydrolysis and product uptake might be coupled. The MFS transporter contains 12 transmembrane-spanning helices (as predicted by Phyre2 (http://www.sbg.bio.ic.ac.uk/phyre2/html/page.cgi?id=index)), with the last C-terminal helix and the attached GH17 domain (Fig. 2e), which would enable the simultaneous cleavage of oligosaccharides and the sugar transported through the MFS. Blast analysis revealed that this fusion is common among marine *Flavobacteriia*, suggesting that such multi-modular transporter-associated enzyme may be a conserved mechanism for boosting laminarin utilization.

In order to examine the molecular basis of substrate specificity the X-ray crystal structure of GH17A was solved (Fig. 2f, see Supplementary Information). Compared to the monomeric GH17 structures, FbGH17A has significant insertions and is larger. The structure of GH17A allows for the deduction of the molecular basis of substrate specificity for the laminarinase. Based on the GH17 complexes obtained for *R. miehei* [32], a model was generated of a laminarin product involving five monomers bound to the catalytic groove, two on the aglycone side, and three on the glycone side (Fig. 2g). The reducing and non-reducing ends of the modeled glycan are free, suggesting the protein can act in the middle of the chain as expected for an endo-acting glycoside hydrolase (see Supplementary Information). Furthermore, given the conformation of the modeled glycan, 6-O-β-glucose branching would be possible only at subsite + 1 and anything further away (+ 3 or − 4). In other words, within the native polysaccharide the enzyme would need a stretch of at least three free β1,3-glucose moieties to act. This structural data supports the observation that GH17 activity on laminarin is bolstered by the action of the debranching enzyme GH30.

### Laminarin stimulates the co-expression of selected peptidases and transporters

**Fig. 3:**
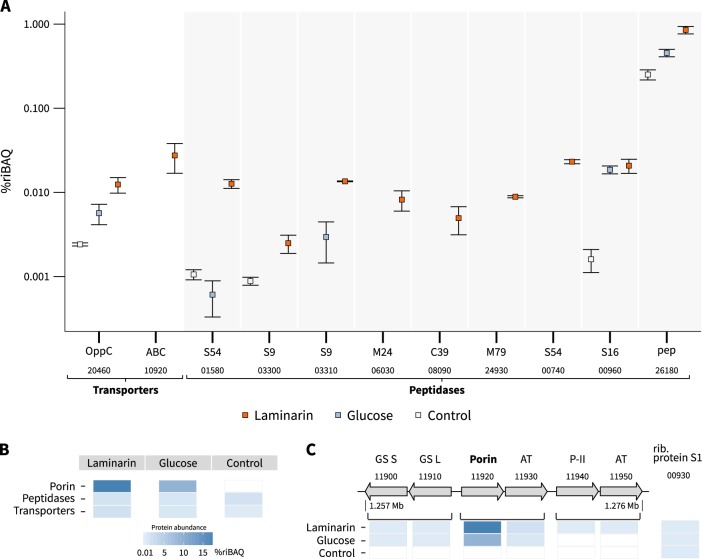
Abundance of peptidases and putative peptide transporter proteins of *Formosa* B. **a** Proteomic signatures of selected proteases and two putative peptide transporters, which showed an increased expression under laminarin conditions. The two laminarin-induced peptidases shown on the right (00960, 26180) were also detected in the metaproteome of a spring bloom in 2009 (see Supplementary Table S8A). Relative protein abundances are depicted as % riBAQ values. The putative peptidase families and the respective locus tags are indicated. The squares represent the mean values of the three replicates for every protein and each substrate. The error bars refer to the standard error of the mean. Proteins that could be detected in at least two out of three independent biological replicates of each substrate condition are shown (for individual replicate numbers see Supplementary Table S2A). **b** Comparison of total protein abundances (in %riBAQ) of peptidases (see Supplementary Table S4), nitrogen-associated transporters and the porin (FORMB_11920) in *Formosa* B under the three investigated substrate conditions (see Supplementary Table S3). **c** Genomic structure of the porin-encoding cluster and the abundance patterns of the corresponding proteins. Brackets indicate putative operons. Protein functions and the respective locus tags are indicated. GS S: glutamate synthase subunit S, GS L: glutamate synthase subunit L, AT: ammonium transporter, P-II: nitrogen regulatory protein P-II

*Formosa* B encodes 69 peptidases in its relatively small genome (2.7 MB). Other laminarin-degrading marine *Bacteroidetes* like *Gramella forsetii* KT0803^T^ (79 peptidases), *Polaribacter* sp. Hel1_85 (84 peptidases), *Jejuia pallidilutea* (58 peptidases), and *Flaviramulus ichthyoenteri* (63 peptidases) show a comparable number of peptidase genes, although their genomes are around twice as large as that of *Formosa* B. Our proteome analysis of *Formosa* B revealed that 41 peptidases are expressed in the presence of laminarin (Supplementary Table S4). Nine of these peptidases showed a significantly higher protein abundance on laminarin in the enriched membrane proteome, compared with glucose or the control culture, or were exclusively found after incubation with laminarin (Fig. 3a and Supplementary Table S3). In addition, a putative peptide ABC transporter ATP-binding protein (FORMB_10920) and a putative oligopeptide permease ABC transporter protein (OppC; FORMB_20460) were detected, which showed a significantly higher abundance under laminarin conditions (Supplementary Table S3). This indicates a coupling of the peptide metabolism with laminarin utilization in *Formosa* B.

An exceptionally high expression with glucose and laminarin was visible for a putative porin (FORMB_11920, 10% riBAQ; Fig. 3b, c), an outer membrane protein, which was not detectable in the control cultivations with peptone (Supplementary Table S3). The porin-encoding gene is located in an operon with a putative ammonium transporter and clusters with several genes involved in nitrogen metabolism, including two putative glutamate synthase genes and an additional supposed ammonium transporter (Fig. 3c). All nitrogen metabolism-related genes in the direct vicinity of the porin-encoding gene were only found to be expressed with glucose and laminarin in peptone-containing cultures in comparison with the peptone-only control culture without these carbon sources (Fig. 3c).

### In situ abundance and relevance of Formosa strain A and B

**Fig. 4:**
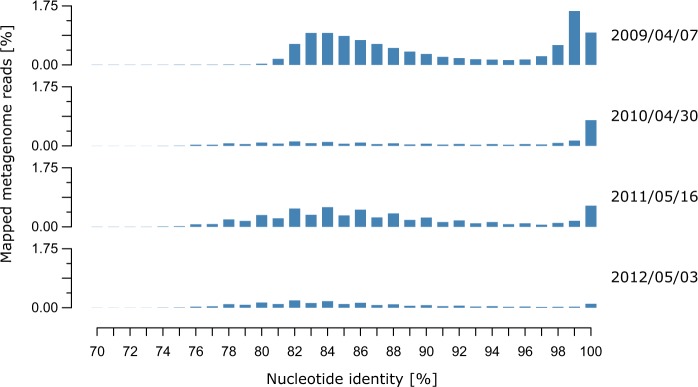
Relative abundance of *Formosa* strain B and related species during four spring phytoplankton blooms indicated by the percentage of metagenomics reads mapped at different nucleotide identities. Reads recruited at ≤ 93% nucleotide identity represent other *Formosa* spp. that are abundant during the spring bloom events at Helgoland, Germany from 2009 until 2012. Dates on the right indicate the four metagenomes (i.e., time points), which produced highest mapping coverage with the *Formosa* strain B genome in their respective years. For a summary of all 44 metagenomes (up to 18 time points per year) and their mapping results see Supplementary Table S5

We investigated the in situ abundance of the *Formosa* strains A and B by recruiting *Formosa* reads from the 44 metagenomes of the years 2009–2012 from Helgoland bacterioplankton samples [18]. At the ≥ 95% average nucleotide identity (ANI) threshold, the strain A and B genomes recruited up to 0.28% and 2.94% of individual metagenomic reads in 2009, 0.04% and 0.99% for 2010, 0.03% and 0.98% for 2011, and 0.02% and 0.21% for 2012 (Supplementary Table S5), respectively. The mapped reads covered up to 91%, 99%, 97%, and 94% of the strain B genome from 2009 to 2012, respectively, and only up to 58% of the strain A genome in 2012 (Fig. 4 and Supplementary Table S5). This suggests that strain B was recurrent and abundant during the spring bloom events, whereas strain A was likely more representative for late summer blooms reaching highest abundances of mapped reads in September 2009. Reads mapped to the *Formosa* strain B genome with 70–93% ANI suggest the presence of other closely related *Formosa* spp. during the spring blooms of 2009 to 2012 that reached up to 6.84%, 1.2%, 5.1%, and 1.8% of the metagenome reads, respectively (Fig. 4). Altogether, these results indicate that strain B is one of the representatives of the recurrent *Formosa* clade during North Sea spring microalgae blooms [17, 18].

### Identification of Formosa-specific enzymes and transporters during microalgal blooms

**Fig. 5:**
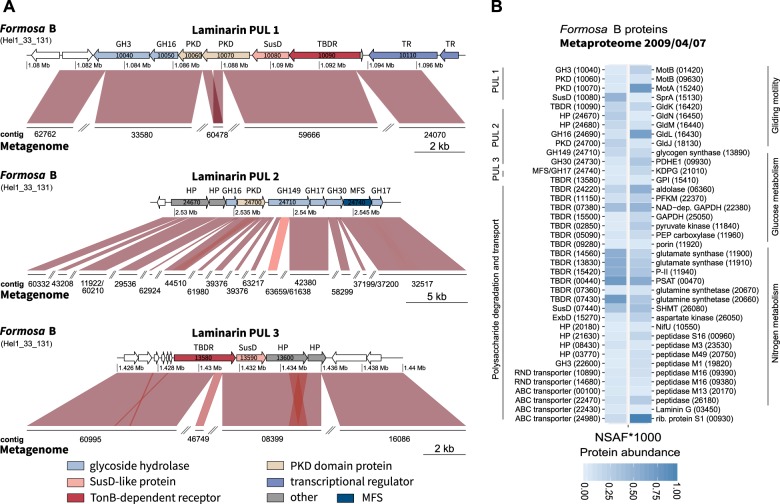
Detection of *Formosa* strain B laminarin PULs in the Helgoland spring bloom metagenome and metaproteome in 2009 [17]. **a** Synteny between the laminarin PULs of *Formosa* sp. Hel1_33_131 and partial PUL sequences in the metagenomes from 2009/04/07. The sequence comparisons were performed with Bl2seq (BLASTn, *E* value 1e-5). Sequence similarities are depicted by red hues for direct comparisons. Darker colors correspond to higher identities. Gene locus tags are subsequent numbers within PULs and are indicated in the figure for most of the genes (for visibility’s sake, gene names of very small genes were omitted). **b** Heatmap of the relative abundance of *Formosa* strain B proteins (displayed as normalized spectral abundance factor values, NSAF*1000) detected in the metaproteome from 07 April 2009. Displayed are selected proteins, which likely play a role in polysaccharide or protein utilization. A highly abundant ribosomal protein of *Formosa* strain B (rib. protein S1, lower right) is also displayed as a reference to illustrate the high abundance of polysaccharide utilization-specific proteins during the bloom condition. Gene locus tag numbers are given in parenthesis. GH, glycoside hydrolase; PKD, PKD-domain containing protein; SusD, SusD-family protein; TBDR, TonB-dependent receptor; HP, hypothetical protein; MFS, major facilitator superfamily; ExbD, subunit of the Ton system for energy transduction; MotB, motor rotation protein; Gld, gliding motility proteins; PDHE, pyruvate dehydrogenase E1 component; KDPG, 2-dehydro-3-deoxyphosphogluconate aldolase; GPI, glucose-6-phosphate isomerase; PKFM, 6-phosphofructokinase; GAPDH, glyceraldehyde 3-phosphate dehydrogenase; P-II, nitrogen regulatory protein P-II; PSAT, phosphoserine aminotransferase; SHMT, serine hydroxymethyltransferase

All three *Formosa* B PULs were completely covered by metagenomic contigs of the spring bloom in 2009 and 2010 (Fig. 5a and Supplementary Tables S6–S7), and partially covered in the metagenomes of 2011 and 2012 (Supplementary Table S7). This illustrates the strong selection pressure imposed by laminarin on this pathway during four consecutive annual spring phytoplankton blooms in the North Sea.

We examined the presence of polysaccharide degradation- and consumption-related proteins of the *Formosa* strains A and B in the in situ metaproteomes of spring blooms in 2009 and 2010 (Supplementary Table S8). The proteome analysis of the planktonic bacterial fraction sampled during the spring bloom on 7 April 2009 uncovered 46 proteins from *Formosa* strain A and 361 proteins from *Formosa* strain B. Remarkably, several marker proteins from the putative laminarin-specific *Formosa* B PULs were highly abundant (Fig. 5b and Supplementary Table S8A) in the metaproteome samples. This analysis identified 13 proteins of the PULs 1, 2, and 3 (see also Supporting Information) and thus indicated that a significant proportion of *Formosa* B’s laminarin PULs were expressed in situ during the spring bloom in 2009. Although the metaproteome analysis of 2010 uncovered fewer proteins from both *Formosa* strains, three marker proteins of PUL 1 from *Formosa* B were detected in the environmental samples (see Supplementary Information and Supplementary Table S8B).

Besides glycoside hydrolases and laminarin-specific transporter proteins, we also identified several *Formosa* B proteins in the environmental metaproteome samples of 2009, which are involved in the central catabolism of the monosaccharide glucose, the product of laminarin hydrolysis (see Fig. 5b, Supplementary Information and Supplementary Table S8A). This includes nearly all glycolytic enzymes as well as a putative glycogen synthase of *Formosa* B. These data indicate that the *Formosa* B strain substantially contributed to laminarin degradation and turnover during a diatom-driven phytoplankton bloom.

In addition, several proteins of *Formosa* B involved in nitrogen metabolism could be detected in the metaproteome analyses of the spring bloom 2009 (Supplementary Table S8A). This includes the putative porin (FORMB_11920), a peptide ABC transporter ATP-binding protein (FORMB_10920), an oligopeptide permease ABC transporter protein (OppC; FORMB_20460), and eight peptidases (Fig. 5b). This underlines a strong coupling of the peptide metabolism with laminarin utilization of *Formosa* B under in situ conditions.

## Discussion

This study provides detailed insights in the adaptations which make *Formosa* strains successful competitors in the early breakdown of organic matter during diatom blooms. Combining comparative in vitro and in situ proteogenomics with biochemical enzyme characterization reveals that the key to this process is the sensing and utilization of laminarin. Our data indicate that this polysaccharide is used in two ways: as a major source of energy, and as a signal molecule, which induces transporters and digestive enzymes to use also other compounds released from the lysis of diatom cells.

The two environmentally relevant *Formosa* strains examined in this study feature streamlined genomes, which are significantly smaller than those of many other marine *Flavobacteriia*. With a lower number of total proteins to synthesize, *Formosa* A and B can dedicate a higher relative proportion of their genomic and proteomic resources to the digestion of laminarin. Their CAZyme repertoire is strongly reduced compared to versatile polysaccharide degraders such as *F. agariphila* [24] and *Zobellia galactanivorans* [35], which were isolated from macroalgae. It is, however, similar to another member of North Sea spring bacterioplankton, *Polaribacter* sp. Hel1_33_49 [7]. In contrast to macroalgae-associated laminarin-degrading bacteria, such as *Z. galactanivorans* [43], neither of the *Formosa* strains possesses a mannitol dehydrogenase, which indicates a specialization of *Formosa* A and B to chrysolaminarin. This type of laminarin lacks mannitol residues and is preferentially produced by diatoms.

We found a specific laminarin protein abundance pattern in *Formosa* B, which differs from the protein expression pattern in presence of the sugar monomer of this polysaccharide, glucose. A similar laminarin-specific control of gene expression was suggested for the marine flavobacterium *G. forsetii* [4]. Interestingly, this laminarin-specific proteome signature of *Formosa* B includes not only the proteins required for laminarin uptake and utilization, but also peptidases and transporters for amino-acid utilization. The *Formosa* cells, upon sensing of laminarin, thus appear to react in two ways: First, they enhance the expression of outer membrane proteins to degrade and rapidly transport the energy molecule laminarin into their periplasm, utilizing the selfish polysaccharide uptake mechanism recently demonstrated for marine *Flavobacteriia* [42]. Second, the abundance of amino-acid and nitrogen metabolism-related proteins is increased to boost the recycling of nitrogen building blocks, which are required for rapid growth of *Formosa* bacteria and become available simultaneously with laminarin upon algal lysis.

**Fig. 6:**
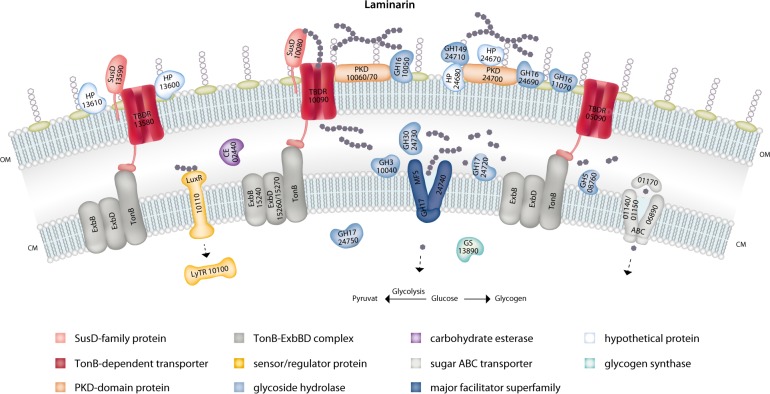
A tentative model of laminarin utilization pathways in *Formosa* B. Protein localizations were predicted in silico according to Romine [46] and were deduced by subproteome analyses (see also Supplementary Table S3). Additional biochemical experiments are required to ascertain this model

*Formosa* strain B possesses an extended repertoire of laminarin-specific enzymes and transporters, which is larger than that of other laminarin-degrading bacteria such as *Polaribacter* sp. Hel1_33_49 [7] or *G. forsetii* [4]. Our subproteome and bioinformatic analyses indicate that many laminarin-degrading enzymes of *Formosa* B are surface-tethered or localized in the periplasmic space and in the cytoplasmic membrane, respectively. The different TBDR, laminarinases, transporters, and additional enzymes combine complementary activities into an efficient laminarin disassembly line for degradation and uptake (Fig. 6). The biochemical experiments presented here support the annotation of the conserved cluster of genes as encoding for a laminarin utilization pathway. Here, two enzymes that are likely residents of the periplasm are shown to work together towards the complete degradation of laminarin in a highly specific manner. The X-ray crystal structure of GH17A reveals the possible molecular determinants of substrate specificity and the propensity of the enzyme to be more active on unbranched laminarin.

The multi-modular protein FORMB_24740 (FbGH17b) combines a glycoside hydrolase (GH17) with a membrane spanning transport protein and may represent an adaptive mechanism for laminarin utilization. The integration of the transport and hydrolysis processes into a single protein could facilitate improved consumption of the sugars by increasing the activity of the fused GH17. Increased activity would also reduce the necessary enzyme copy number, and thereby resource consumption for synthesis of this protein. To our knowledge, such a transporter-CAZyme-fusion has not been described for other bacteria as yet, but its conservation in nature suggests that this could provide a significant benefit.

The exceptionally strong accumulation of a putative porin in glucose- and laminarin-controlled cultures and the co-induction of the 12 surrounding genes of this porin-encoding genomic cluster, all of which play a role in nitrogen metabolism, could indicate a function of this transporter protein in the uptake of peptides as nitrogen and amino-acid source. Endo-acting proteases might degrade proteins released by lysed microalgae into peptides, which are then imported through the porin into the periplasm. An efficient capture of these peptides with a highly abundant porin system might be especially useful in the highly diffusive marine environment. With laminarin and glucose as easily metabolizable carbon sources, such a strategy could be crucial for a balanced carbon and nitrogen diet.

Members of the phylum *Bacteroidetes* are primary degraders of microalgal polysaccharides during phytoplankton blooms, and are therefore key players in marine carbon cycling. However, underlying enzymatic mechanisms and adaptations that drive the specialization of these highly competitive bacteria remain obscure. We reveal and prove in this study the specific activity and ecological niche of two abundant marine *Bacteroidetes* strains in complex microbial communities during diatom-driven phytoplankton blooms. Our results show an extraordinary degree of specialization for the *Formosa* strains A and B, which enable these marine *Bacteroidetes* to successfully compete for laminarin against a multitude of other laminarin-degrading microbes in bloom situations [1, 4, 7, 44, 45]. Our data furthermore indicate that fast growth on beta-glucans such as laminarin requires a balanced diet that also includes nitrogen sources like peptides. The induction of several cell wall-associated peptidases and peptide-specific transporters in *Formosa* B during growth on laminarin suggests that these bacteria pursue a complex uptake strategy, which encompasses both sugars and nitrogen compounds. This may make marine *Flavobacteriia* so successful in their diffusion-open environment.

## Electronic supplementary material

Supporting Information

SI Figure S6

SI Table S1

SI Table S2A

SI Table S2B

SI Table S2C

SI Table S3

SI Table S4

SI Table S5

SI Table S6

SI Table S7

SI Table S8AB
