# Supplementary material for: Adaptive mechanisms that provide competitive advantages to marine bacteroidetes during microalgal blooms
Source: ISME J. 2018 Jul 30;12(12):2894–906. doi: 10.1038/s41396-018-0243-5 (PMC6246565; doi:10.1038/s41396-018-0243-5)
Supplement: Supplementary file 2 — SI Figure S6 [file 41396_2018_243_MOESM2_ESM.pdf]

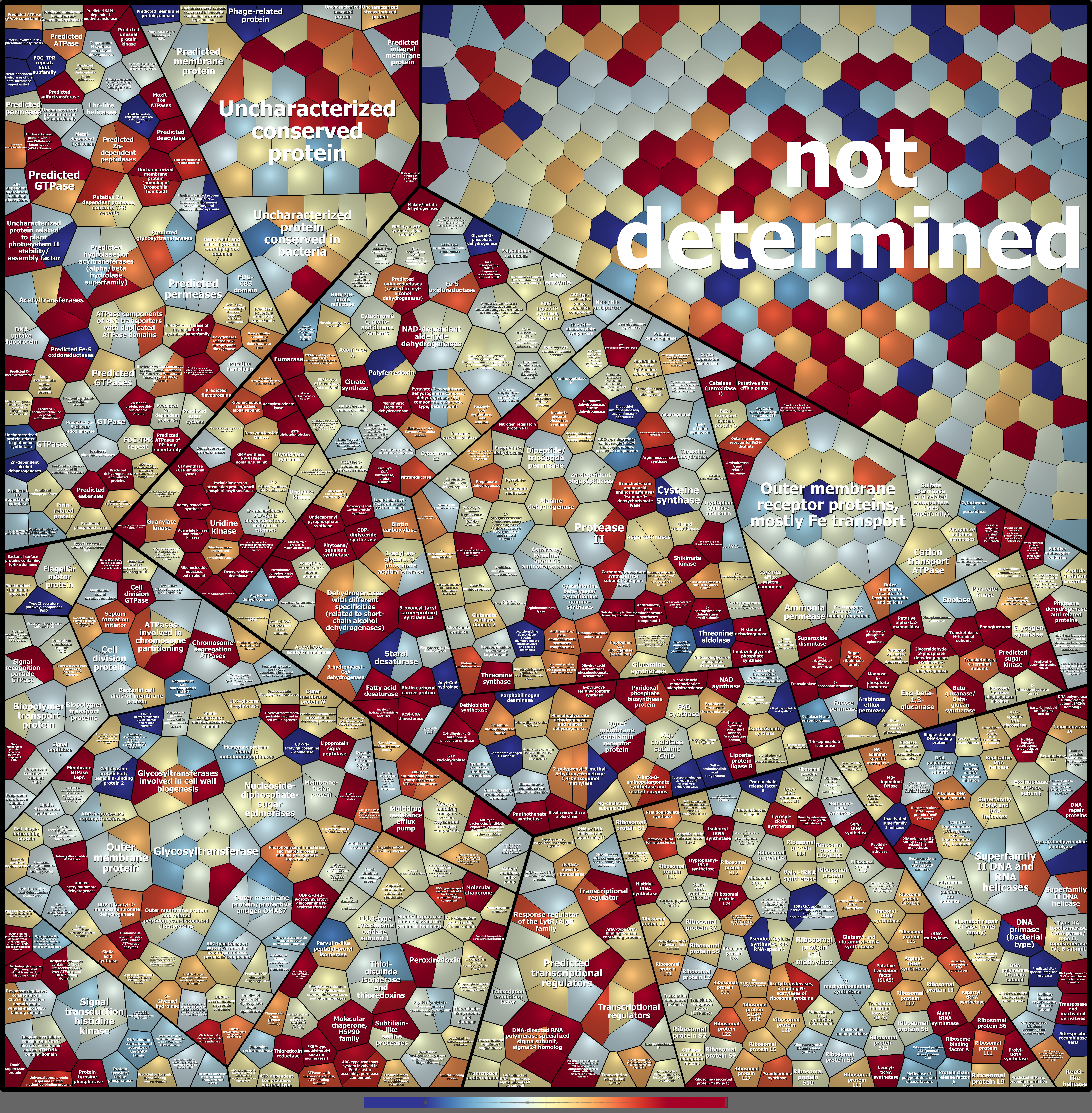
not determined
Uncharacterized conserved protein
Uncharacterized protein conserved in bacteria
Phage-related protein
Predicted membrane protein
Predicted integral membrane protein
Predicted ATPase
Predicted GTPase
Predicted GTPases
GTPases
GTPase
Predicted permeases
Predicted permease
Acetyltransferases
ATPase components of ABC transporters with duplicated ATPase domains
Predicted Fe-S oxidoreductases
Predicted hydrolases or acyltransferases (alpha/beta hydrolase superfamily)
Uncharacterized protein related to plant photosystem II stability/assembly factor
Predicted Zn-dependent peptidases
Predicted deacylase
Lhr-like helicases
MoxR-like ATPases
FOG: TPR repeat, SEL1 subfamily
FOG: CBS domain
FOG: TPR repeat
Predicted esterase
Predicted flavoproteins
Putative hemolysin
Predicted glycosyltransferases
Flagellar motor protein
Cell division GTPase
Cell division protein
ATPases involved in chromosome partitioning
Chromosome segregation ATPases
Septum formation initiator
Signal recognition particle GTPase
Biopolymer transport protein
Biopolymer transport proteins
Glycosyltransferases involved in cell wall biogenesis
Nucleoside-diphosphate-sugar epimerases
Glycosyltransferase
Outer membrane protein
Outer membrane protein/protective antigen OMA87
Multidrug resistance efflux pump
Membrane-fusion protein
Lipoprotein signal peptidase
Signal transduction histidine kinase
Molecular chaperone, HSP90 family
Molecular chaperone
Peroxiredoxin
Thiol-disulfide isomerase and thioredoxins
Subtilisin-like serine proteases
Thioredoxin reductase
Parvulin-like peptidyl-prolyl isomerase
Response regulator of the LytR/AlgR family
Predicted transcriptional regulators
Transcriptional regulators
Transcriptional regulator
DNA-directed RNA polymerase specialized sigma subunit, sigma24 homolog
Fumarase
Citrate synthase
Polyferredoxin
Aconitase A
NAD-dependent aldehyde dehydrogenases
Fe-S oxidoreductase
Malic enzyme
Uridine kinase
Guanylate kinase
Uridylate kinase
Thymidylate synthase
Dehydrogenases with different specificities (related to short-chain alcohol dehydrogenases)
Sterol desaturase
Fatty acid desaturase
Biotin carboxylase
Acyl-CoA hydrolase
Dipeptide/tripeptide permease
Zn-dependent oligopeptidases
Protease II
Cysteine synthase
Alanine dehydrogenase
Threonine synthase
Threonine aldolase
Glutamine synthetase
Outer membrane cobalamin receptor protein
Mg-chelatase subunit ChlD
FAD synthase
NAD synthase
Lipoate-protein ligase B
Pyridoxal phosphate biosynthesis protein
Porphobilinogen deaminase
Outer membrane receptor proteins, mostly Fe transport
Cation transport ATPase
Ammonia permease
Superoxide dismutase
Enolase
Glycogen synthase
Exo-beta-1,3-glucanase
Beta-glucanase/Beta-glucan synthetase
Catalase (peroxidase I)
Superfamily II DNA and RNA helicases
Superfamily II DNA helicase
DNA primase (bacterial type)
Ribosomal protein L11 methylase
Pseudouridylate synthases, 23S RNA-specific
Ribosomal protein S2
Ribosomal protein S6
Ribosomal protein S3
Ribosomal protein L9
Ribosomal protein S8
Ribosomal protein L13
Ribosomal protein S10
