# Supplementary material for: Adaptive mechanisms that provide competitive advantages to marine bacteroidetes during microalgal blooms
Source: ISME J. 2018 Jul 30;12(12):2894–906. doi: 10.1038/s41396-018-0243-5 (PMC6246565; doi:10.1038/s41396-018-0243-5)
Supplement: Supplementary file 1 — Supporting Information [file 41396_2018_243_MOESM1_ESM.pdf]

Supporting information

Adaptive mechanisms that provide competitive advantages to marine Bacteroidetes during microalgal blooms

Frank Unfried[1,2,3], Stefan Becker[2,4], Craig S. Robb[2,4], Jan-Hendrik Hehemann[2,4], Stephanie Markert[1,3], Stefan E. Heiden[1], Tjorven Hinzke[1,3], Dörte Becher[3,5], Greta Reintjes[2], Karen Krüger[2], Burak Avcı[2], Lennart Kappelmann[2], Richard L. Hahnke[2,6], Tanja Fischer[2], Jens Harder[2], Hanno Teeling[2], Bernhard Fuchs[2], Tristan Barbeyron[7], Rudolf I. Amann[2], Thomas Schweder[1,3,#]

[1] Pharmaceutical Biotechnology, University Greifswald, Germany

[2] Max Planck Institute for Marine Microbiology, Bremen, Germany

[3] Institute of Marine Biotechnology, Greifswald, Germany

[4] MARUM, Center for Marine Environmental Sciences at the University of Bremen, Bremen, Germany

[5] Institute for Microbiology, University Greifswald, Germany

[6] DSMZ, Braunschweig, Germany

[7] National Center of Scientific Research/Pierre and Marie Curie University Paris 6, UMR 7139 Marine Plants and Biomolecules, Station Biologique de Roscoff, Roscoff, Bretagne, France

[#]To whom correspondence should be addressed: Thomas Schweder, University Greifswald, Pharmaceutical Biotechnology, Felix-Hausdorff-Straße 3, 17487 Greifswald, Germany

Tel: +49 3834 420 4212; Fax: +49 3834 420 4238; e-mail: schweder@uni-greifswald.de

This Supporting Information PDF file contains:

Supplementary Methods and Results with references
Supplementary Figures S1 – S11
Supplementary Tables S9

The Supplementary Tables S1 – S8 are available as separate Excel files.

Supplementary Methods

Bioinformatic analyses

Sequence alignments between genome and metagenome sequences were performed using Bl2seq (BLASTn, E-value 1e-5) (Camacho *et al.*, 2009). Sequence data and the BLAST comparison files were drawn with the R package genoPlotR (Guy *et al.*, 2010) version 0.8.4 and edited in Inkscape version 0.91. BLAST results were automatically edited, so that short hits contained in longer hits and hits with a bitscore below 100 were removed.

For the estimation of the *in situ* abundance of the *Formosa* strains A and B during algal spring blooms quality filtered reads from 44 metagenomes (Supplementary Table S5) were mapped on the respective genomes. Reads were quality filtered and adapters trimmed using bbduk.sh (v. bbmap-35.14) from the BBtools package (Bushnell, 2016) (https://sourceforge.net/projects/bbmap/) with the following settings: ktrim=r, k=28, mink=12, hdist=1, tbo=t, tpe=t, qtrim=rl, trimq=20, minlength=100. Reads were then mapped on the genomes using bbmap.sh (BBtools package) with the following settings, using a nucleotide identity threshold of ≥95%: minid=0.95, idfilter=0.95. Sequence alignment map files (SAM) were further processed to binary format (BAM) using samtools (v. 1.2) (Li *et al.*, 2009). Unmapped (-F 4) and low quality mapped reads (-q <10), and reads that were detected as PCR duplicates (VALIDATION_STRINGENCY=LENIENT) using picard tools (v. 1.119) (http://broadinstitute.github.io/picard/) were removed. The number of mapped reads and coverage information was subsequently calculated using pileup.sh (BBtools package). For detection of closely related *Formosa* species in 4 representative metagenomes (2009/04/07, 2010/04/30, 2011/05/16, 2012/05/03), reads were mapped to the *Formosa* strain B genome using bbmap.sh with nucleotide identity threshold of ≥70% (settings: minid=0.76, idfilter=0.70). Mapped reads were directly extracted from SAM files and are shown as the fraction of reads mapped to the *Formosa* B genome compared to the total number reads from the metagenome [%].

The amino acid sequence of the catalytic domains from putative glycoside hydrolases of family GH16 from *Formosa* sp. Hel1_33_131 were blasted (BlastP) against all characterized GH16 enzymes from the CAZy database. Hits with an identity of ≥25% and a query coverage of ≥80% were selected for a MUSCLE alignment (Edgar, 2004). Afterwards, the alignment was used to construct a maximum likelihood tree using bootstrap values. Phylogenetic and molecular evolutionary analyses were conducted using MEGA version 6 (Tamura *et al.*, 2013).

Determination of laminarin uptake

For the determination of laminarin uptake by *Formosa* B, cells were grown in HaHa medium (Hahnke *et al.*, 2015) with laminarin (2 g L^{-1}) and harvested during exponential growth. The cells were then inoculated (1:10) in minimal medium and 35 µmol fluorescently labelled (FLA) laminarin was added (Arnosti, 2003). The cells were sampled for visualization before the inoculation and after 5, 10 and 30 min. Cells were fixed using formaldehyde (1%) for 1 h at room temperature and subsequently filtered onto polycarbonate (0.2 µm pore size) filters. The cells were counterstained using DAPI and visualized using super-resolution structured illumination microscopy (SR-SIM) as described in detail by Reintjes *et al.* (Reintjes *et al.*, 2017).

Subproteome fractionation

To extract the soluble intracellular proteome and the enriched membrane-associated proteome, cell pellets of *Formosa* strain B were resuspended in TE buffer (10 mM Tris-HCl pH 7.5, 10 mM EDTA pH 8.0) with Roche 'cOmplete Protease Inhibitor'. Cells were disrupted by sonication (3 x 30 s) and cell debris was removed by centrifugation (8.000 x *g*, 4 °C, 10 min). Soluble intracellular proteins and membrane-associated proteins were separated via ultracentrifugation (100.000 x *g*, 4 °C, 60 min). Purification of the membrane-bound proteome was performed as described by Kabisch *et al.* (2014). To enrich the soluble extracellular proteome, the culture supernatants were precipitated with 10% TCA (trichloroacetic acid) overnight at 4 °C (Antelmann *et al.*, 2001), and precipitated proteins were extracted by centrifugation (9.500 x *g*, 4 °C, 60 min) and washed with ice-cold ethanol (99.9%) before dissolving them in 8 M urea/2 M thiourea.

From each subproteome fraction and triplicate, 25 µg of protein were loaded on a 10% 1D-SDS-polyacrylamide gel and separated according to their molecular weight for 75 min at 150 V. After fixing with ethyl acetate and Coomassie G-250 staining (Candiano *et al.*, 2004), the proteins were in-gel digested for 16 h using trypsin as described by Heinz *et al.* (2012). Peptides were eluted in an ultrasonic bath for 15 min and subsequently desalted using ZipTip columns (Millipore, Billerica, MA, USA) according to the manufacturer's guidelines.

Cloning procedures

The gene encoding the putative laminarinase FbGH17A (locus tag FORMB_24720) was amplified from genomic DNA of *Formosa* sp. Hel1_33_131 (GenBank accession number GenBank: CP017260.1) (Hahnke *et al.*, 2015). The following primers were designed to amplify the coding regions without the signal peptide-encoding sequences using Gibson assembly (Gibson *et al.*, 2009):

FbGH17Aforward: CAGCGGCCTGGTGCCGCGCGGCAGCCATAAAAATAAGCAATCAAATACG,

FbGH17Areverse: CTCAGTGGTGGTGGTGGTGGTGCTCGAGTTAATGGTTCTTTGTGATTTC.

They include ~30 bp overhangs complementary with the pET28a+ vector, for Gibson assembly (Gibson *et al.*, 2009), which results in an N-terminal hexahistidine tag in the recombinant protein. The PCR followed standard PCR amplification protocols and the Gibson cloning kit (NEB) was used according to the manufacturer's instructions. Colonies of positive clones were identified by colony PCR using T7. The vector from a positive clone was purified from 2 mL Luria-Bertani (LB) broth cultures using the QIAprep Spin Miniprep kit (Qiagen). For protein expression, the vector was used to transform *Escherichia coli* BL21 (DE3) (NEB). The FaGH17B gene (locus tag FORMB_24740) was synthesized and cloned into a pET28a+ vector by the company GenScript. Unlike the two other proteins, the vector was transformed in *E. coli* Rosetta (DE3) (Merck-Millipore). The cells were stored at -80 °C until further use.

Enzyme overexpression, refolding and purification

Protein expression was conducted in ZYP autoinduction medium (Studier, 2005), which was inoculated with an overnight preculture at 37 °C in LB medium supplemented with 100 µg mL^{-1} kanamycin. Cultures were grown for 3 days at 20 °C in ZYP medium with 100 µg mL^{-1} kanamycin, with rotation at 150 rpm. Cells were harvested by centrifugation at 4,500 x *g* for 25 min at 4 °C, and the pellet was stored at -20 °C until further use. After thawing, the cells were suspended in 15 mL sucrose solution (20 mM Tris-HCl, 25% [w/v] sucrose). 30 mg of lysozyme was added and the cells were incubated for 10 min at room temperature and constant stirring. Afterwards 30 mL of deoxycholate solution (20 mM Tris-HCl pH 7.5, 1% [w/v] deoxycholate, 100 mM NaCl, 1% Triton X-100) and 0.2 mL of 1 M $MgSO_4$ were added. The highly viscous solution was liquefied by adding 10 mg of DNase (Sigma) at room temperature. The resulting lysate was centrifuged at 16,000 x *g* for 45 min at 4 °C.

The supernatant of FbGH17A and FbGH30 was loaded onto a 5-mL HiTrap IMAC HP column (GE Healthcare), which was charged with one column volume (CV) of 500 mM $NiSO_4$ and equilibrated with 5 CVs of IMAC buffer A (20 mM Tris-HCl pH 8, 500 mM NaCl, 20 mM imidazole). After sample injection, the column was washed with IMAC buffer A (15 CVs), and the protein was eluted with 5 CVs of a linear gradient from IMAC buffer A to IMAC buffer B (20 mM Tris-HCl pH 8, 500 mM NaCl, 500 mM imidazole), at a flow rate of 5 mL min^{-1}, at 20 °C. The 1-mL fractions were analyzed by SDS-PAGE. Fractions corresponding to a band at the expected size were pooled and concentrated in an ultrafiltration stirred cell (Amicon, Merck-Millipore), on a polyether-sulfone membrane with a 10-kDa cutoff value (Merck-Millipore). An aliquot of 1 to 2 mL was loaded onto a 120-mL HiPrep 16/60 Sephacryl S-200 HR column that had been equilibrated previously with 3 CVs of SEC buffer (20 mM Tris-HCl pH 7.5, 500 mM NaCl) at 20 °C. The protein was eluted with 1 CV of SEC buffer, and the fractions were analyzed by SDS-PAGE. Fractions with a single band at the expected size and at an elution time corresponding to monomeric

or dimeric protein were combined and concentrated. The concentration and hydrodynamic radius were determined using a BioSpectrometer (Eppendorf) and dynamic light scattering (DLS).

Since FbGH17B was expressed insoluble, it had to be refolded *in vitro* (adapted from Qi *et al*. (2015)). After bacterial cell lysis, the resulting pellet contained the denatured protein. The pellet was resuspended in 30 mL washing buffer (20 mM Tris-HCl pH 8, 300 mM NaCl, 1 mM EDTA, 1% [v/v] Triton X-100, 1 M Urea), followed by centrifugation at 16,000 x *g* for 15 min and 4 °C. This washing step was repeated three times. The pellet was resuspended in 10 ml refolding buffer (20 mM Tris-HCl pH 8, 1 M Urea) and stored overnight at -20 °C. After thawing, the solution was centrifuged as described before. To remove the urea, the solution was dialyzed overnight against 1 L SEC buffer and using a Spectra/Por 1 dialysis membrane with a 6-8 kDa cutoff (Spectrum Labs). The reservoir was kept at 4 °C and stirred permanently. To remove the precipitate resulting from the dialysis, the protein solution was centrifuged again. Afterwards, it was ready to be purified via IMAC in the same way as the other proteins. The 1-mL fractions were analyzed by SDS-PAGE but in contrast to the other proteins, they were pure enough to omit the SEC purification. Fractions corresponding to a band at the expected size were pooled and concentrated. In order to remove the imidazole, the protein was additionally applied on a 5-mL HiTtrap desalting column (GE Healthcare). The procedure was executed according to the manufacturer's instructions and the column was equilibrated with SEC buffer.

Hydrolysis of laminarin

Laminarin from *Laminaria digitata* (0.1% [w/v]; Sigma) was hydrolyzed over the course of 60 min at 37 °C with 100 nM purified enzyme (~5 µg mL^{-1} of FbGH30, FbGH17A, or FbGH17B) in 50 mM MOPS buffer at pH 7. Aliquots of 100 µL were taken at 0 s, 5 min, 10 min, 20 min, 40 min and 60 min. Each reaction was stopped by boiling the sample for 5 min at 100 °C.

Debranching and purification of laminarin

Laminarin was debranched with FbGH30. 100 mg laminarin was hydrolyzed overnight under the conditions mentioned above. The reaction was stopped by boiling the sample for 5 min at 100 °C. Precipitated protein was removed by filtration through 0.2 µm Costar Spin-X Filters (Corning). Afterwards, the debranched high molecular laminarin was separated from glucose by size exclusion chromatography using a HiTrap Desalting column (GE Healthcare) according to the manufacturer's instructions. The column was equilibrated and the sample was eluted with Milli-Q water. The water was evaporated overnight in a vacuum concentrator at 45 °C and constant rotation (Eppendorf).

Michaelis-Menten-Kinetics

The kinetic parameters of the investigated enzymes acting on native and debranched laminarin from *L. digitata* (Sigma) were determined using 100 nmol of the enzyme in a reaction mixture of 600 µL over 10 min at 37 °C and in MOPS buffer at pH 7. Seven different laminarin concentrations were measured in triplicates: 0% (w/v), 0.05%, 0.1%, 0.2%, 0.4%, 0.8%, 1.6%. 100 µL aliquots were taken every 2 min and the reaction was stopped by boiling the sample for 5 min at 100 °C. The amount of released reducing ends was measured by the PAHBAH reducing sugar assay (Moretti and Thorson, 2008). One mL of a freshly prepared 9:1 mixture of reagent A (0.3 M 4-hydroxybenzhydrazide, 0.6 M HCl) and reagent B (48 mM trisodium citrate, 10 mM $CaCl_2$, 0.5 M NaOH) was added to each aliquot and the mixture was heated for 5 min at 100 °C. Absorbance was determined at 410 nm using a BioSpectrometer (Eppendorf).

High Performance Anion Exchange Chromatography with Pulsed Amperometric Detection (HPAEC-PAD)

An HPAEC-PAD system was applied for qualitative product analysis. An ICS-5000+ (Dionex) with electrochemical detection on the gold working electrode and a pH reference electrode (Ag/AgCl) was used. The detector wave form was: E1=100 mV (T=0.4 s), E2=-2000 mV (T=0.42 s), E3=600 mV (T=0.43 s) and E4=-100 mV (T=0.5 s). A 25 µL sample loop was used on a Dionex CarboPac PA100 analytical column (2×250 mm) coupled with a Dionex CarboPac PA100 guard column (2×50mm). Chromatography and detection were conducted at 25 °C. A mixed sugar standard solution consisting of 1 µg mL^{-1} Glucose (Sigma), laminaribiose, laminaritriose, laminaritetraose, laminaripentaose and laminarihexaose (all from Megazyme) was used as reference. Eluent 1 consisted of 0.15 M NaOH (HPLC grade, VWR) and eluent 2 of 0.15 M NaOH and 1 M sodium acetate (HPLC grade, Sigma). All eluents were dissolved in Milli-Q water, degassed with helium for at least 10 min and kept pressurized after connecting the bottles to the system. Eluent 2 was filtered through a 0.2 µm nylon filter membrane. Separation was achieved by a linear gradient course of two mobile eluents (from 100% eluent 1 to 50% eluent 1 and 50% eluent 2) over a duration of 19.5 min, followed by an increase of the eluent 2 concentration to 70% within 6 s and a linear gradient to 100% eluent 2 over 114 s immediately afterwards. Finally, the concentration of eluent 1 was increased back to 100% over 30 s. The entire sequence was conducted at a flow rate of 0.25 mL/min. The column was equilibrated with eluent 1 for 3 min between each sample.

Phylogenetic analysis

All available *Formosa*-related 16S rRNA sequences from SILVA SSURef v.128 database (Quast *et al.*, 2013) were loaded into ARB v.6.1 (Ludwig *et al.*, 2004) and consistently aligned using SILVA Incremental Aligner (SINA) (Pruesse *et al.*, 2012). Phylogenetic tree reconstructions were done with the ARB internal programs (i) for maximum likelihood method RAxML v.8 (Stamatakis, 2014) using the 'GTRGAMMA' substitution model and 'thorough tree search' option activated, (ii) the ARB neighbor joining program using the Jukes-Cantor correction and (iii) the 'ARB_PARSIMONY' maximum parsimony method for global and local optimizations. All treeing algorithms were run on sequences filtered with and without a 30% and 50% positional conservation filter for all *Flavobacteriia*. A consensus tree was built from these trees following the recommendation by Peplies *et al.* (Peplies *et al.*, 2008). Therefore, bootstrap values cannot be given, and branches indicating ≤1% distance should be regarded as uncertain.

Supplementary Results

Metaproteomic identification of Formosa-specific enzymes and transporters during microalgal blooms
For the metaproteome analysis, we generated combined *in silico* databases of all potential peptides using the recorded metagenome data from the spring blooms in 2009 (Teeling *et al.*, 2012) and 2010 (Teeling *et al.*, 2016), respectively, as well as the predicted protein sequences of the *Formosa* A (Hel3-A1_48) and B (Hel1_33_131) genomes. The combined search of the measured peptide spectra from the spring bloom samples taken on April 7th 2009 against this database yielded several marker proteins of *Formosa* B's PUL 1, including the glycoside hydrolase GH3 (FORMB_10040), a PKD domain protein (FORMB_10070), the TBDR protein (FORMB_10080), and the SusD-like protein (FORMB_10090) (Figure 2B and Supplementary Table S5A). In addition, we detected the PUL 2-encoded *Formosa* B proteins glycoside hydrolase GH2 (FORMB_24670), two putative GH16 (FORMB_24680, FORMB_24690), a GH30 (FORMB_24730), a PKD domain protein (FORMB_24700), a hypothetical protein (FORMB_24710), and the MFS transporter protein (FORMB_24740). Furthermore, also the TBDR protein (FORMB_13580) of *Formosa* B's PUL 3 could be identified in the environmental samples (Figure 5B and Supplementary Table S8A). Altogether, this analysis provided evidence that a significant proportion of *Formosa* strain B's putative laminarin PUL-encoded proteins were expressed *in situ* during the spring bloom in 2009. Furthermore, the metaproteome analysis also revealed the three *Formosa* B marker proteins of PUL 1, FORMB_10060, FORMB_10080, and FORMB_10090, in the environmental samples of 2010 (Supplementary Table 8B).

Additional *Formosa* B proteins that were detected in the environmental metaproteome samples of 2009 are involved in the central catabolism of the laminarin-specific monosaccharide glucose (Figure 5B and Supplementary Table S8A). Nearly all glycolysis-related enzymes were detected. This includes glucose-6-phosphate isomerase (EC 5.3.1.9, FORMB_15410), fructose-bisphosphate aldolase (EC 4.1.2.13, FORMB_06360), triosephosphate isomerase (EC 5.3.1.1, FORMB_18610), glyceraldehyde-3-phosphate dehydrogenase (GAPDH, EC 1.2.1.12, FORMB_25050; NAD-dependent GAPDH, EC 1.2.1.12, FORMB_22380), 2,3-bisphosphoglycerate-independent phosphoglycerate mutase (EC 5.4.2.11, FORMB_06010), and pyruvate kinase (EC 2.7.1.40, FORMB_11840). In addition, the pyruvate dehydrogenase E1 component beta subunit (EC 1.2.4.1, FORMB_09930), the anaplerotic enzyme phosphoenolpyruvate (PEP) carboxylase (EC 4.1.1.31, FORMB_11960) and a putative glycogen synthase (EC 2.4.1.21, FORMB_13890) of *Formosa* B could be identified in the metaproteome samples of the spring bloom 2009 (Figure 5B and Supplementary Table S8A). These data demonstrate that the *Formosa* B strain substantially contributed to laminarin degradation and turnover during a diatom-driven phytoplankton bloom.

Furthermore, we identified glycolytic marker proteins of *Formosa* strain A in the metaproteome samples (Supplementary Table S8AB). These include, for example, the fructose-bisphosphate aldolase class II (EC 4.1.2.13, FORMA_07040), and the pyruvate dehydrogenase E1 component beta subunit (EC 1.2.4.1, FORMA_09500), during the spring bloom 2009, and the glyceraldehyde-3-phosphate dehydrogenase (EC 1.2.1.12, FORMA_16240) in 2010 environmental samples.

Laminarin-induced proteins of Formosa B

With laminarin as growth substrate, the TonB-dependent-receptor protein FORMB_10090 belonged to the most strongly induced proteins within the laminarin PULs (Figure 1C). Moreover, this TBDR protein was one of the most abundant proteins of the entire membrane-enriched proteome in this study (0.8% riBAQ; Supplementary Table S2A) and was also particularly abundant in the bacterioplankton metaproteome during the spring bloom in April 2009 (Figure 5B and Supplementary Table S8A). Furthermore, the TBDR protein FORMB_10090 was likewise identified together with the PUL 1 - specific proteins FORMB_10080 (SusD-like protein) and FORMB_10060 (PKD domain protein) in the metaproteome of the spring bloom in May 2010 (Figure 5B and Supplementary Table S8B). This highlights the importance of this polysaccharide uptake protein in glycan utilization. In the control experiments with glucose or peptone, this receptor protein (FORMB_10090) was also detectable, albeit at significantly lower levels. Such a weak constitutive expression pattern was also observed for other PUL-encoded proteins and might be explained by a sensory function of these proteins (Hehemann *et al.*, 2012, Kabisch *et al.*, 2014, Thomas *et al.*, 2013). The constitutive, low basal

expression of selected proteins, which are involved in initial polysaccharide degradation and uptake steps, would allow bacteria to scan their environment for potential substrates without having to maintain the entire machinery for polysaccharide turnover.

A phylogenetic analysis of 9 putative laminarinases of *Formosa* B indicated a clear affiliation to the GH16 family for three of the enzymes (Supplementary Figure S9). Two of the respective genes are located in the laminarin-inducible PULs 1 and 2 (Figure 1C). The laminarin-induced protein machinery of *Formosa* B includes additional proteins, which are not organized in PULs, such as the putative laminarinase (FORMB_24690) of the GH16 family (Supplementary Figure S9A and Supplementary Table S3). This enzyme belonged to the most highly expressed proteins in the proteome during growth with laminarin but was not detectable with glucose or in the control culture (Supplementary Figure S9B). We furthermore detected a GH5 protein (FORMB_08760), which is encoded in an operon with three other proteins, all of which were also upregulated in the presence of laminarin (Supplementary Table S3). A putative polysaccharide deacetylase (FORMB_02440), which possesses a NodB-like domain of the carbohydrate esterase family 4 (CE4), was also found to be induced by laminarin (Supplementary Table S3). The second domain of this multi-modular enzyme seems to be a glycoside hydrolase of the GHL13 family. Elevated protein abundance under laminarin conditions was also observed for an ABC-transporter protein (FORMB_12400), which is encoded in an operon with a putative aspartate-semialdehyde dehydrogenase. It is interesting to note that the glycolytic enzymes glyceraldehyde-3-phosphate dehydrogenase (FORMB_25050) and the pyruvate kinase (FORMB_11840) were also found to be induced in *Formosa* B cultures with laminarin, which indicates an upregulation of this part of the glycolytic pathway under laminarin conditions. Furthermore, the increased level of the phosphoenolpyruvate carboxylase (FORMB_11960) during the growth on laminarin could ensure the replenishment of oxaloacetate in the citric acid cycle to support the metabolic flux through this pathway and to thus boost anabolic and energy generation processes.

Beside the PUL-encoded TBDRs we identified FORMB_05090 as an additional laminarin-induced TBDR protein (Supplementary Figure S10 and Supplementary Table S3). We detected further TBDR proteins, the expression of which was upregulated by both laminarin and glucose (Supplementary Figure S10). This includes FORMB_02790 und FORMB_02850, which are located in an operon with two hypothetical proteins in a proposed mannan PUL (Supplementary Figure S3). The glucose and laminarin-induced TBDR protein FORMB_07380 is located in an operon with a SusD-like protein of unknown function. The gene encoding the upregulated TBDR protein FORMB_15500 is located in an operon with a gene encoding a hypothetical membrane protein, which contains a putative galactose-binding domain. These TBDRs were also detected in the metaproteome samples of the spring bloom in 2009. It is interesting to note that the gene of the highly abundant TBDR protein FORMB_15420, which could also be identified in the metaproteome samples (Figure 5B), clusters genomically together with the

gene of the glucose-6-phosphate isomerase (FORMB_15410) and a hypothetical protein of unknown function.

Subproteome analyses

The bioinformatic prediction of the putative cellular localization of laminarin-induced proteins of *Formosa* B as suggested in Figure 6 was based on the strategy proposed by Romine (Romine, 2011) (Supplementary Table S3). The computational analysis of specific domains and putative protein sorting signals was combined with the subproteome analyses of the enriched soluble intracellular, membrane-associated and extracellular protein fractions to improve confidence in our protein location predictions. A PorSS signal sequence of the novel type IX protein secretion system (Sato *et al.*, 2010) was predicted for the laminarinase GH16 (FORMB_11070) as well as for a number of hypothetical proteins, suggesting an extracellular localization of these enzymes (see Supplementary Table S3). These analyses also suggested a surface-tethered localization of the laminarinase GH16 FORMB_10050, the hypothetical protein FORMB_24710, the GHs FORMB_13600 and FORMB_24680 or the PKD-domain proteins of the laminarin specific PULs. The multi-modular MFS-GH17 fusion protein FORMB_24740 with 12 membrane-spanning domains was enriched in the membrane proteome fraction.

Our subproteome enrichment procedure does not provide an exclusive separation of intracellular, membrane-associated and secreted proteins from each other, but, as an enrichment, gives indications on the actual protein localization. The proposed model of the laminarin utilization machinery in *Formosa* B illustrated in Figure 6 is, therefore, a working model and requires further in-depth analyses in the future to prove specific protein interactions and their subcellular organization.

Structural analysis of GH17A

In order to examine the molecular basis of substrate specificity the X-ray crystal structure of GH17A was solved to a resolution of 2.6Å by molecular replacement using a GH17 from the fungus *Rhizomucor miehei* (Qin *et al.*, 2015). The structure was modelled without gaps from residues 38 to 430, the C-terminus (Supplementary Table S9). Within the crystal lattice, the protein is found as a dimer of trimers where a C-terminal loop blocks the active site of the adjacent monomer (Supplementary Figure S11). The structure consists of a modified $(\alpha/\beta)_8$ fold whereas other characterized members of GH17 are smaller and unmodified (Varghese *et al.*, 1994). Compared to the monomeric GH17 structures, FbGH17A has significant insertions and is larger (Figure 2F). The closest structurally characterized GH17 homolog from *R. miehei* (Qin *et al.*, 2015) shares an overall Cα RMSD of 1.85 Å and a structurally aligned sequence identity of 23%. The insertions of FbGH17A are found in two main places. First, the loop β3-

α3 is longer and is found adjacent to the active site. Second, the helix α6 is extended and the C-terminal loop wraps around this extension to reach into the adjacent active site (Figure 2F). The latter insertions appear to be involved in oligomerization as opposed to catalysis as they are far away from the active site.

The Supplementary Tables S1 – S8 are available as separate Excel files. Legends are given below:

Supplementary Table S1. Summary of the phylogeny-guided CAZyme annotations for *Formosa* strain A and B in comparison to the macro algae-associated strain *Formosa agariphila* KMM 3901T.

Supplementary Table S2. Overview of *Formosa* B subproteome analyses performed in this study during growth on laminarin, glucose and chitin. (**A**) Enriched membrane proteome, (**B**) enriched intracellular proteome, and (**C**) enriched proteome from the supernatant of the cultures (extracellular proteome).

Supplementary Table S3. List of laminarin-induced *Formosa* B proteins and their predicted subcellular localization. The prediction is based on *in silico* analyses according to Romine (Romine, 2011) and subproteome analyses (Supplementary Tables S2A-C). IP: soluble intracellular proteome, MP: enriched membrane proteome, EP: enriched extracellular proteome. The following bioinformatic tools were used: General prediction of protein localization: PsortB (http://www.psort.org/psortb/index.html) and SecretomeP (http://www.cbs.dtu.dk/services/SecretomeP/); Prediction of Sec signal peptides: LipoP (http://www.cbs.dtu.dk/services/LipoP/) and SignalP (http://www.cbs.dtu.dk/services/SignalP/); prediction of proteins with membrane-spanning domains: TMHMM (http://www.cbs.dtu.dk/services/TMHMM/) and BOMP (http://services.cbu.uib.no/tools/bomp) (Berven *et al.*, 2004).

Supplementary Table S4. List of all peptidases of *Formosa* B detected in the proteome analyses. The prediction of putative peptidases was supported by *in silico* analyses based on MEROPS 11.0 (https://www.ebi.ac.uk/merops/submit_searches.shtml). Relative protein abundances of all proteins in the three investigated subproteomes are given in %riBAQ.

Supplementary Table S5. Percentage of metagenomic reads mapped to the *Formosa* strain A and B genomes at different nucleotide identities. Quality filtered reads from 44 metagenomes from phytoplankton blooms of four consecutive years were mapped to the respective genomes. Reads were quality filtered and adapters trimmed using bbduk.sh (v. bbmap-35.14) from the BBtools package

(Bushnell, 2016). Reads recruited at ≤ 93% nucleotide identity represent other *Formosa* spp. that were abundant during the bloom events.

Supplementary Table S6. Identity and Bitscore values of the sequence comparison depicted in Figure 3 of the synteny analysis with the laminarin PULs of *Formosa* sp. strain Hel1_33_131 (*Formosa* B) and partial PUL sequences in the metagenomes from the spring bloom at Helgoland in 2009 (Teeling *et al.*, 2012).

Supplementary Table S7. Laminarin-specific, PUL-encoded genes of *Formosa* B as detected in the metagenomes of four consecutive annual spring blooms from 2009 until 2012. green: consecutive contigs; red: overlapping contigs; (): large gap (>300 nt); nd: not detectable

Supplementary Table S8. Relative abundance of *Formosa* A and B proteins in metaproteome samples taken in 2009 (**A**) **and 2010** (**B**). Water samples taken at *Kabeltonne* Helgoland were filtered to enrich pelagic marine bacteria and their metagenome was sequenced (Teeling *et al.*, 2012). All protein-coding genes were included in a metaproteome database, which - for this study - was supplemented with the sequenced genomes of *Formosa* strains A and B. Redundancies between the three datasets were removed by clustering with CD-Hit (at 100% identity across entire sequence length of the shorter protein). The Helgoland metaproteome samples (taken 2009/04/07 and 2010/05/04) were subjected to MS/MS analysis in an Orbitrap Velos mass spectrometer in technical duplicates and the resulting spectra were searched against this combined database. Search results were filtered to give a <1% protein FDR (false discovery rate) and <1% peptide FDR. All proteins, which were identified in either of the two replicates based on database entries from the *Formosa* genomes, are displayed, as well as such hits from the metaproteome database, which are 100% identical to *Formosa* proteins (total of 38 proteins for this sample). Column A lists the source of the protein sequence in the database used (Formosa A: from *Formosa* A genome, Formosa B: from *Formosa* B genome, metaproteome: from metagenome). Average NSAF% values give the abundance of each protein relative to all proteins detected in the same sample.

Supplementary Table S9. Crystallographic data and refinement statistics for FbGH17A

Data collection	
X-ray source	DESY P11
Wavelength (Å)	1.0332
Space group	P2
Unit cell a, b, c (Å)	93.34 149.14 107.22
Unit cell α, β, γ (˚)	90 103.35 90
Resolution range, (Å)	104.32-2.60 (2.65-2.60)
R_{merge}	0.20 (0.766)
Completeness (%)	99.7 (99.9)
Redundancy	5.2 (5.4)
$<I/\sigma(I)>$	6.9 (2.6)
No. of Reflections	453483 (23840)
No. Unique	87396 (4421)
Mosaicity	0.08
Refinement	
R_{work}/R_{free} (%)	20.5/23.9
No. Of Atoms	19699
Protein	18825
Calcium	2
Water	872
***B* factors**	
Overall	26.10
Protein	26.29
Calcium	41.43
Water	19.33
**R.m.s. deviations**	
Bond Lengths (Å)	0.013
Bond Angles (°)	1.668
Ramachandran statistics (%)	
Favored	98.3
Allowed	1.7
Outliers	0.0
PDB accession code	6FCG

Supplementary Figures

Supplementary Figure S1. Phylogenetic consensus tree of the *Formosa* clade. Accession numbers are given at the end of each twig. The two new *Formosa* strains are marked in red. 16S rRNA gene clone sequences retrieved from Helgoland Roads are shown in green. The numbers in brackets indicate the number of grouped sequences. The pink box indicates the specificity of the 16S rRNA-targeted probe FORM181B for Hel1_33_131 (*Formosa* strain B) and closely related clones.

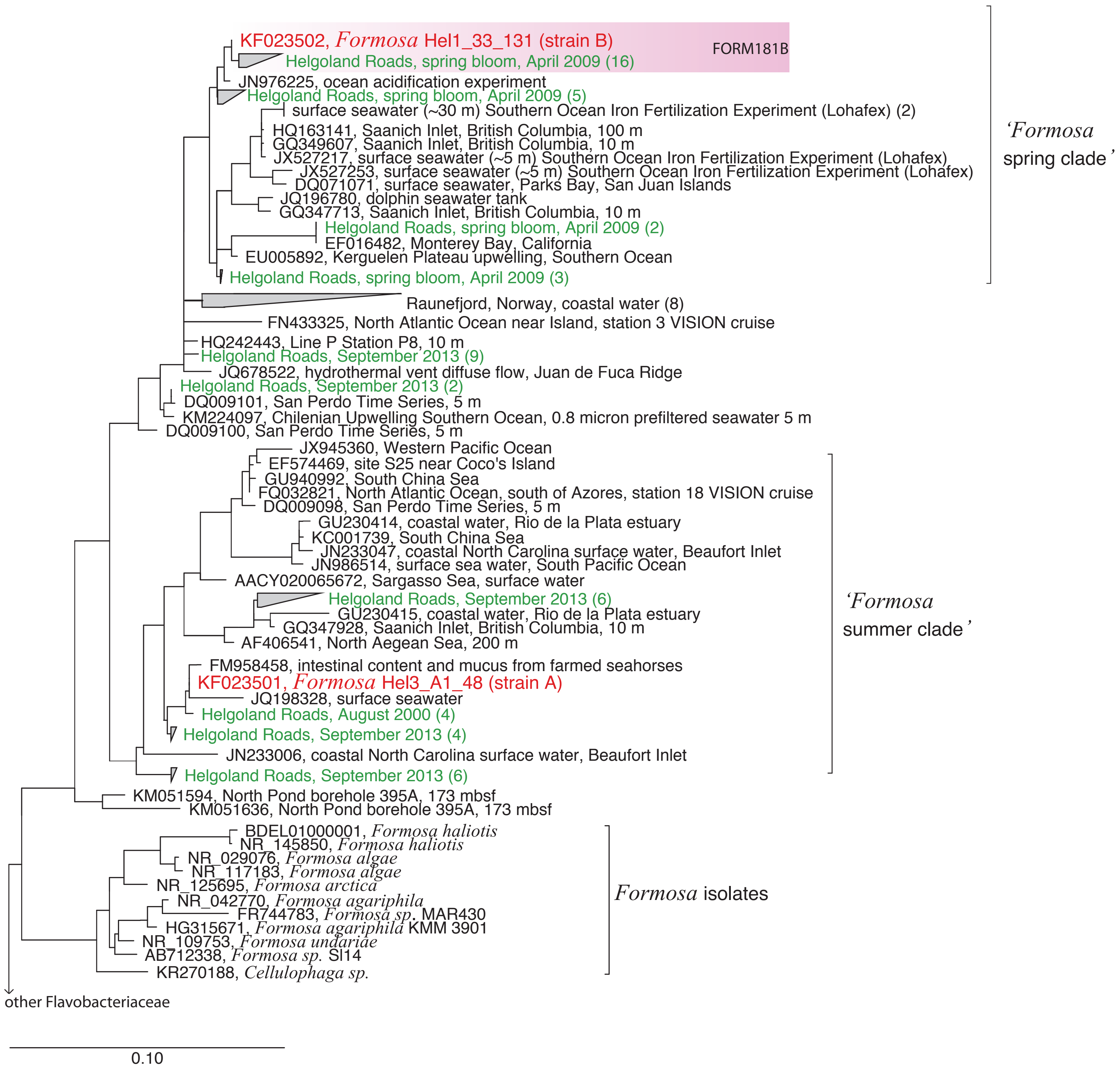
KF023502, Formosa Hel1_33_131 (strain B)
FORM181B
Helgoland Roads, spring bloom, April 2009 (16)
JN976225, ocean acidification experiment
Helgoland Roads, spring bloom, April 2009 (5)
surface seawater (~30 m) Southern Ocean Iron Fertilization Experiment (Lohafex) (2)
HQ163141, Saanich Inlet, British Columbia, 100 m
GQ349607, Saanich Inlet, British Columbia, 10 m
JX527217, surface seawater (~5 m) Southern Ocean Iron Fertilization Experiment (Lohafex)
JX527253, surface seawater (~5 m) Southern Ocean Iron Fertilization Experiment (Lohafex)
DQ071071, surface seawater, Parks Bay, San Juan Islands
JQ196780, dolphin seawater tank
GQ347713, Saanich Inlet, British Columbia, 10 m
Helgoland Roads, spring bloom, April 2009 (2)
EF016482, Monterey Bay, California
EU005892, Kerguelen Plateau upwelling, Southern Ocean
Helgoland Roads, spring bloom, April 2009 (3)
'Formosa spring clade'
Raunefjord, Norway, coastal water (8)
FN433325, North Atlantic Ocean near Island, station 3 VISION cruise
HQ242443, Line P Station P8, 10 m
Helgoland Roads, September 2013 (9)
JQ678522, hydrothermal vent diffuse flow, Juan de Fuca Ridge
Helgoland Roads, September 2013 (2)
DQ009101, San Perdo Time Series, 5 m
KM224097, Chilenian Upwelling Southern Ocean, 0.8 micron prefiltered seawater 5 m
DQ009100, San Perdo Time Series, 5 m
JX945360, Western Pacific Ocean
EF574469, site S25 near Coco's Island
GU940992, South China Sea
FQ032821, North Atlantic Ocean, south of Azores, station 18 VISION cruise
DQ009098, San Perdo Time Series, 5 m
GU230414, coastal water, Rio de la Plata estuary
KC001739, South China Sea
JN233047, coastal North Carolina surface water, Beaufort Inlet
JN986514, surface sea water, South Pacific Ocean
AACY020065672, Sargasso Sea, surface water
Helgoland Roads, September 2013 (6)
GU230415, coastal water, Rio de la Plata estuary
GQ347928, Saanich Inlet, British Columbia, 10 m
AF406541, North Aegean Sea, 200 m
'Formosa summer clade'
FM958458, intestinal content and mucus from farmed seahorses
KF023501, Formosa Hel3_A1_48 (strain A)
JQ198328, surface seawater
Helgoland Roads, August 2000 (4)
Helgoland Roads, September 2013 (4)
JN233006, coastal North Carolina surface water, Beaufort Inlet
Helgoland Roads, September 2013 (6)
KM051594, North Pond borehole 395A, 173 mbsf
KM051636, North Pond borehole 395A, 173 mbsf
BDEL01000001, Formosa haliotis
NR_145850, Formosa haliotis
NR_029076, Formosa algae
NR_117183, Formosa algae
NR_125695, Formosa arctica
NR_042770, Formosa agariphila
FR744783, Formosa sp. MAR430
HG315671, Formosa agariphila KMM 3901
NR_109753, Formosa undariae
AB712338, Formosa sp. SI14
KR270188, Cellulophaga sp.
Formosa isolates
other Flavobacteriaceae
0.10

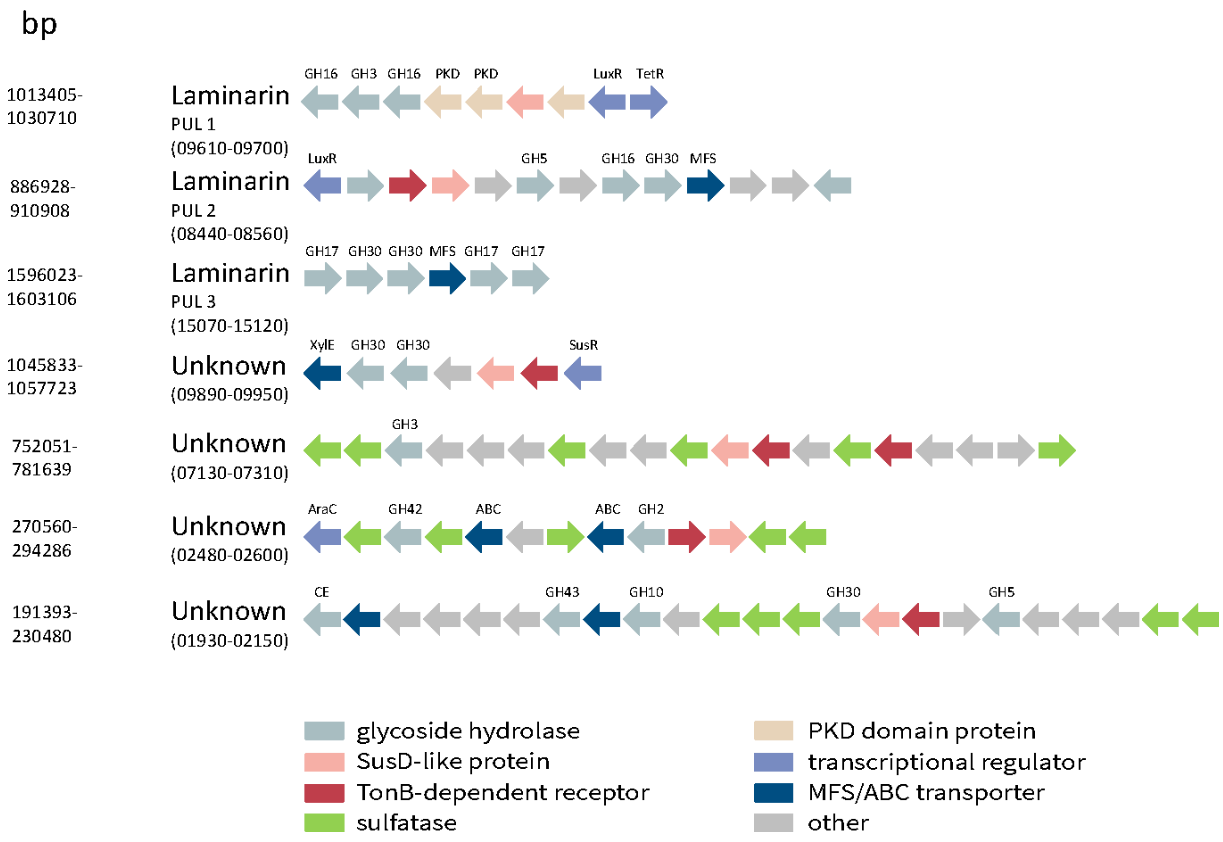

Supplementary Figure S2. PUL organization in *Formosa* A. TBDR, TonB-dependent receptor; SusD, SusD-family protein; PKD, PKD-domain containing protein; GH, glycoside hydrolase; MFS, major facilitator superfamily; HP, hypothetical proteins; CE, carbohydrate esterase; ABC, ABC transporter protein

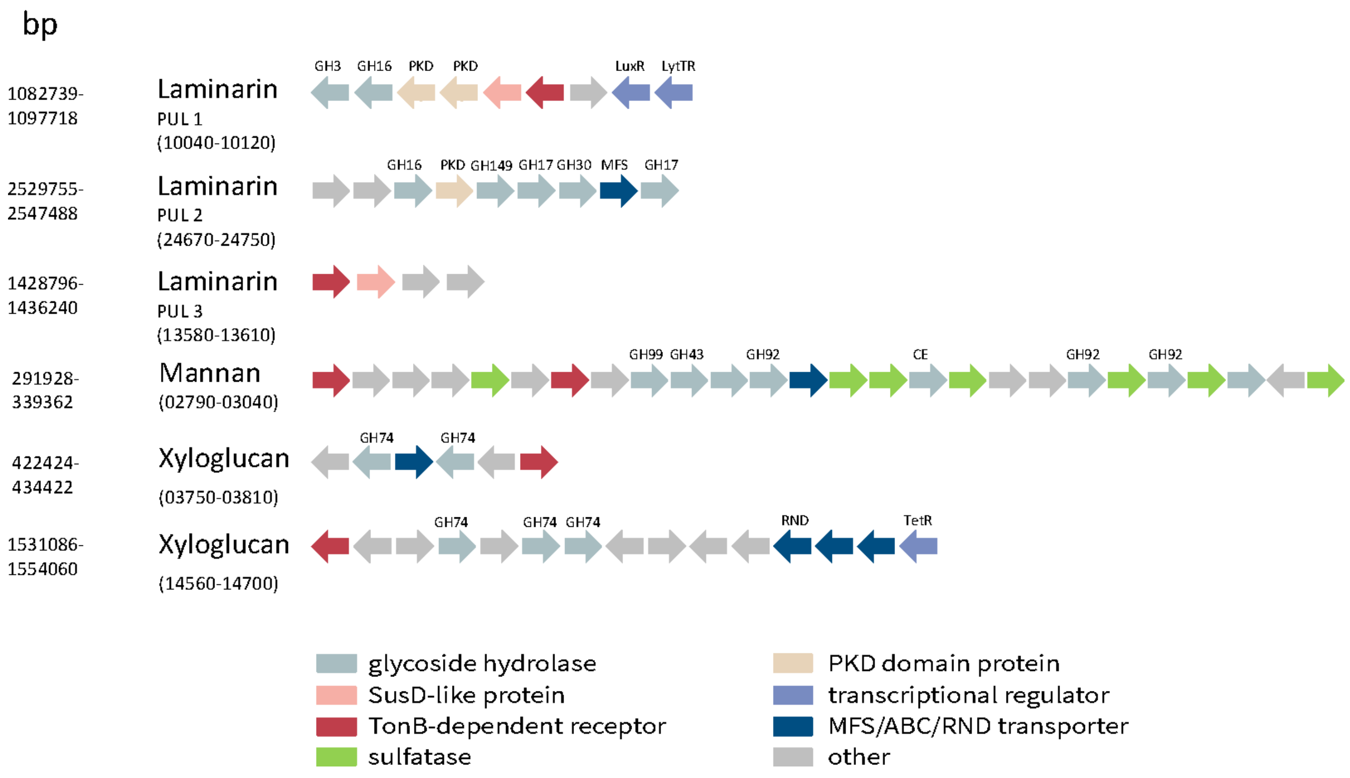

Supplementary Figure S3. PUL organization in *Formosa* B. TBDR, TonB-dependent receptor; SusD, SusD-family protein; PKD, PKD-domain containing protein; GH, glycoside hydrolase; MFS, major facilitator superfamily; HP, hypothetical proteins; CE, carbohydrate esterase; RND, resistance-nodulation-division family transporters

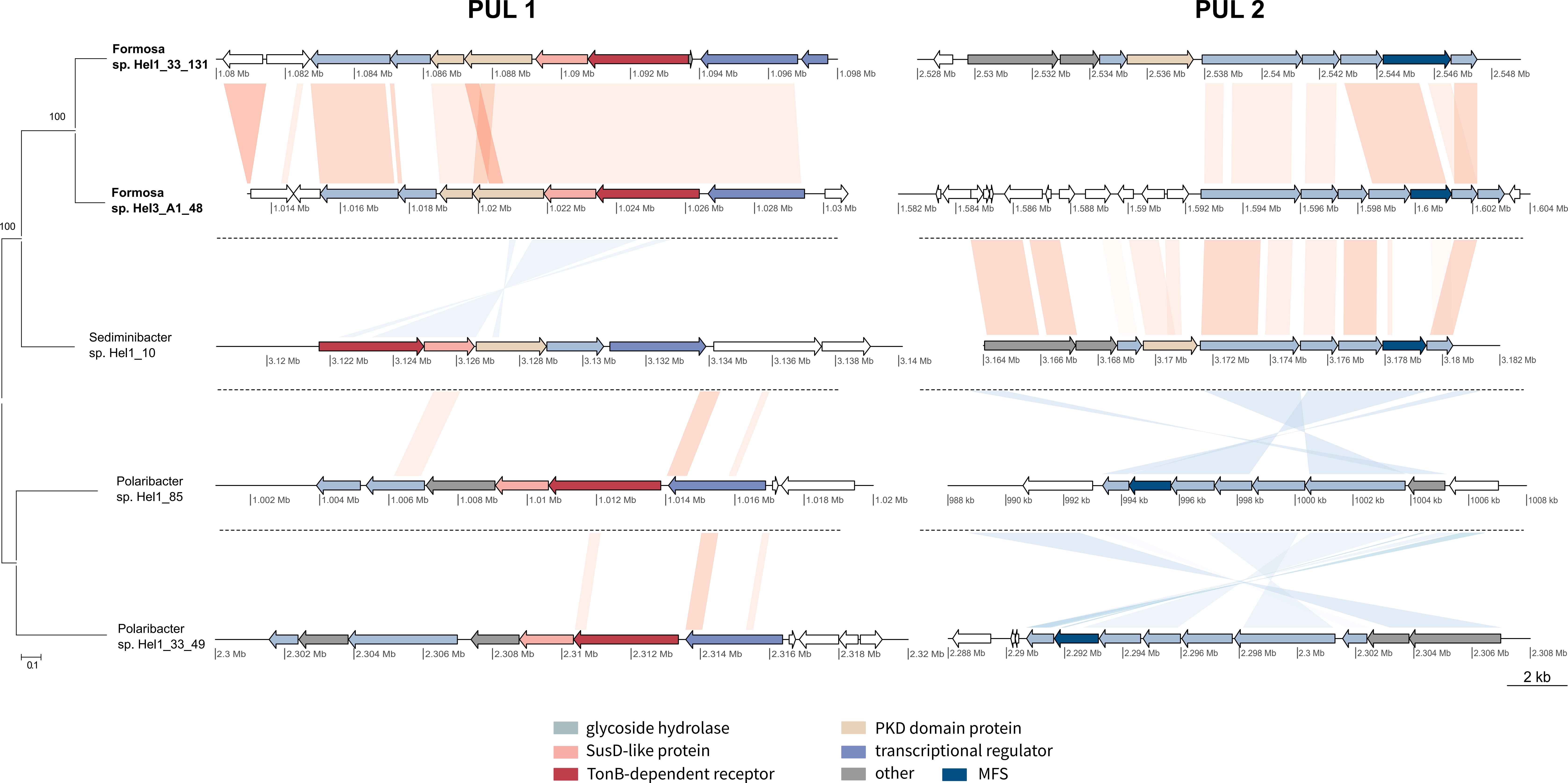
PUL 1
PUL 2
Formosa sp. Hel1_33_131
Formosa sp. Hel3_A1_48
Sediminibacter sp. Hel1_10
Polaribacter sp. Hel1_85
Polaribacter sp. Hel1_33_49
100
100
0.1
1.08 Mb 1.082 Mb 1.084 Mb 1.086 Mb 1.088 Mb 1.09 Mb 1.092 Mb 1.094 Mb 1.096 Mb 1.098 Mb
2.528 Mb 2.53 Mb 2.532 Mb 2.534 Mb 2.536 Mb 2.538 Mb 2.54 Mb 2.542 Mb 2.544 Mb 2.546 Mb 2.548 Mb
1.014 Mb 1.016 Mb 1.018 Mb 1.02 Mb 1.022 Mb 1.024 Mb 1.026 Mb 1.028 Mb 1.03 Mb
1.582 Mb 1.584 Mb 1.586 Mb 1.588 Mb 1.59 Mb 1.592 Mb 1.594 Mb 1.596 Mb 1.598 Mb 1.6 Mb 1.602 Mb 1.604 Mb
3.12 Mb 3.122 Mb 3.124 Mb 3.126 Mb 3.128 Mb 3.13 Mb 3.132 Mb 3.134 Mb 3.136 Mb 3.138 Mb 3.14 Mb
3.164 Mb 3.166 Mb 3.168 Mb 3.17 Mb 3.172 Mb 3.174 Mb 3.176 Mb 3.178 Mb 3.18 Mb 3.182 Mb
1.002 Mb 1.004 Mb 1.006 Mb 1.008 Mb 1.01 Mb 1.012 Mb 1.014 Mb 1.016 Mb 1.018 Mb 1.02 Mb
988 kb 990 kb 992 kb 994 kb 996 kb 998 kb 1000 kb 1002 kb 1004 kb 1006 kb 1008 kb
2.3 Mb 2.302 Mb 2.304 Mb 2.306 Mb 2.308 Mb 2.31 Mb 2.312 Mb 2.314 Mb 2.316 Mb 2.318 Mb 2.32 Mb
2.288 Mb 2.29 Mb 2.292 Mb 2.294 Mb 2.296 Mb 2.298 Mb 2.3 Mb 2.302 Mb 2.304 Mb 2.306 Mb 2.308 Mb
2 kb
glycoside hydrolase
SusD-like protein
TonB-dependent receptor
PKD domain protein
transcriptional regulator
other
MFS

Supplementary Figure S4. The laminarin-specific PULs 1 and 2 in *Formosa* B and A. Gene organization of these PULs is conserved among other marine *Flavobacteriaceae* as exemplified. The PUL structures and their synteny to the *Formosa* B PULs are arranged according to the position of their PUL-specific TBDR proteins in the phylogenetic tree depicted on the left of this figure. The phylogenetic analysis was done by the Maximum Likelihood method based on the JTT matrix-based model (Jones *et al.*, 1992). The tree with the highest log likelihood (-8848.5785) is shown. The tree is drawn to scale, with branch lengths measured in the number of substitutions per site. The analysis involved five amino acid sequences. All positions containing gaps and missing data were eliminated. There were a total of 954 positions in the final dataset. Evolutionary analyses were conducted in MEGA6 (Tamura *et al.*, 2013). Amino acid sequences of the following genes were used to generate this tree: FORMB_10090 (*Formosa* sp. Hel1_33_131), FORMA_09680 (*Formosa* sp. Hel3_A1_48), WP_007807822.1 (*Sediminibacter* sp. Hel1_10), PHEL85_0899 (*Polaribacter* sp. Hel1_85), PHEL49_2085 (*Polaribacter* sp. Hel1_33_49).

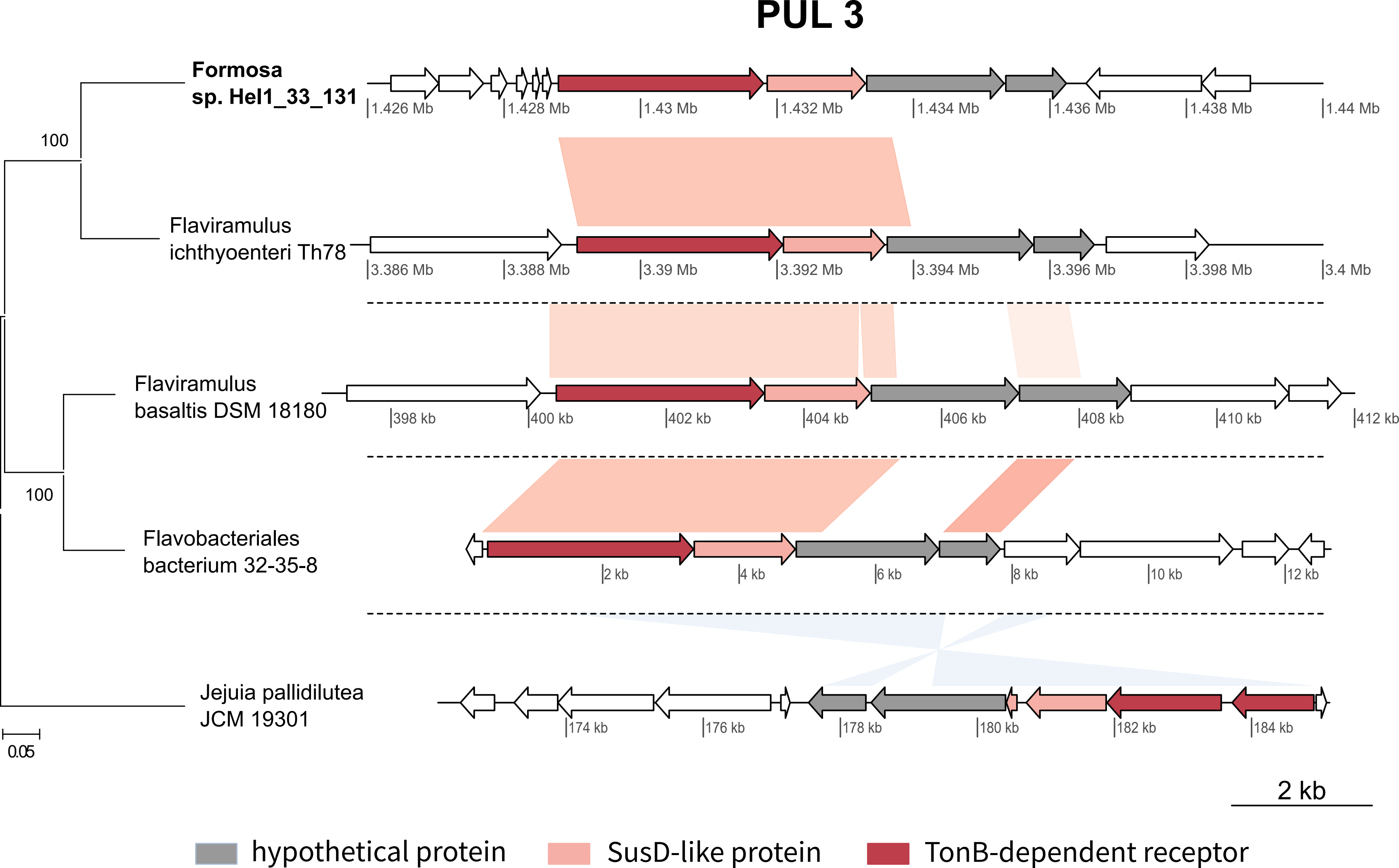
PUL 3
Formosa sp. Hel1_33_131
1.426 Mb
1.428 Mb
1.43 Mb
1.432 Mb
1.434 Mb
1.436 Mb
1.438 Mb
1.44 Mb
100
Flaviramulus ichthyoenteri Th78
3.386 Mb
3.388 Mb
3.39 Mb
3.392 Mb
3.394 Mb
3.396 Mb
3.398 Mb
3.4 Mb
Flaviramulus basaltis DSM 18180
398 kb
400 kb
402 kb
404 kb
406 kb
408 kb
410 kb
412 kb
100
Flavobacteriales bacterium 32-35-8
2 kb
4 kb
6 kb
8 kb
10 kb
12 kb
Jejuia pallidilutea JCM 19301
174 kb
176 kb
178 kb
180 kb
182 kb
184 kb
0.05
2 kb
hypothetical protein
SusD-like protein
TonB-dependent receptor

Supplementary Figure S5. *Formosa* B PUL 3. The third laminarin-specific PUL of *Formosa* B (PUL 3) shows high synteny to other *Flavobacteriaceae*, which do not possess PUL 1 and 2. The PUL structures and their synteny to the *Formosa* B PUL 3 are arranged according to the position of their PUL-specific TBDR proteins in the phylogenetic tree depicted on the left of this figure. The phylogenetic analysis was done by the Maximum Likelihood method based on the JTT matrix-based model (Jones *et al.*, 1992). The tree with the highest log likelihood (-3670.6338) is shown. The tree is drawn to scale, with branch lengths measured in the number of substitutions per site. The analysis involved 5 amino acid sequences. All positions containing gaps and missing data were eliminated. There were a total of 549 positions in the final dataset. Evolutionary analyses were conducted in MEGA6 (Tamura *et al.*, 2013). Amino acid sequences of the following genes were used to generate this tree: FORMB_10090 (*Formosa* sp. Hel1_33_131), RG22_RS14790 (*Flaviramulus ichthyoenteri* Th78), SAMN05428642_102383 (*Flaviramulus basaltis* DSM 18180), OYX28435.1 (*Flavobacteriales* bacterium 32-35-8), JCM19301_3833 (*Jejuia pallidilutea* JCM 19301).

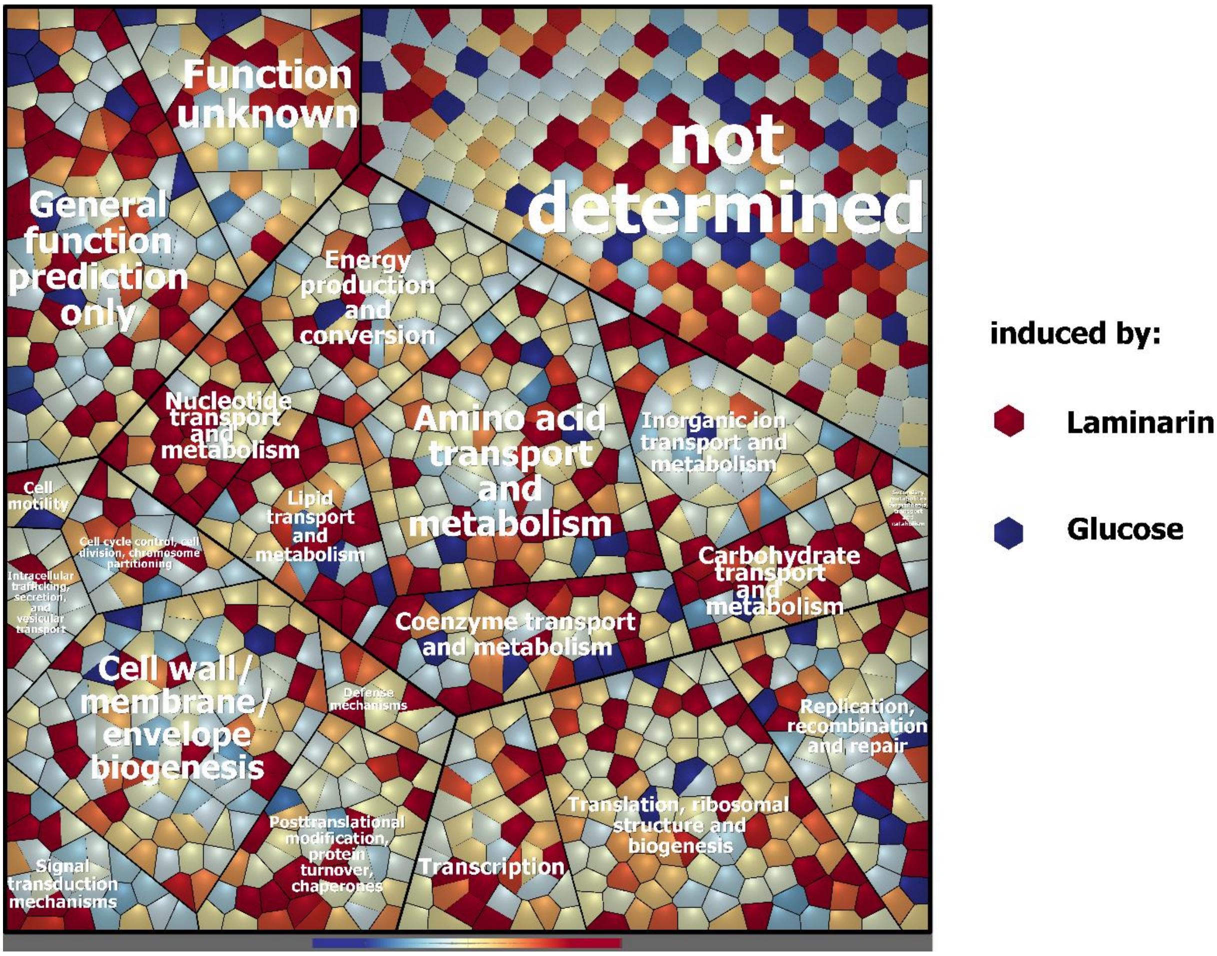

Supplementary Figure S6. Voronoi tree map comparing *Formosa* B protein abundance patterns during growth on laminarin vs. growth on glucose. The color code reflects the calculated log2 ratios of protein abundances under these two substrate conditions. Mean NSAF values (normalized spectral abundance factors) from three biological replicates of the enriched membrane proteome were used for the calculation of expression ratios (fold change values). Functional protein categories were classified with Prophane (Schneider *et al.*, 2011) and the tree map was created using Paver (www.decodon.com/paver.html). A high-resolution image showing the predicted functions of the individual detected proteins in detail is available as separate supplementary file "Supplementary Figure S6 – high resolution".

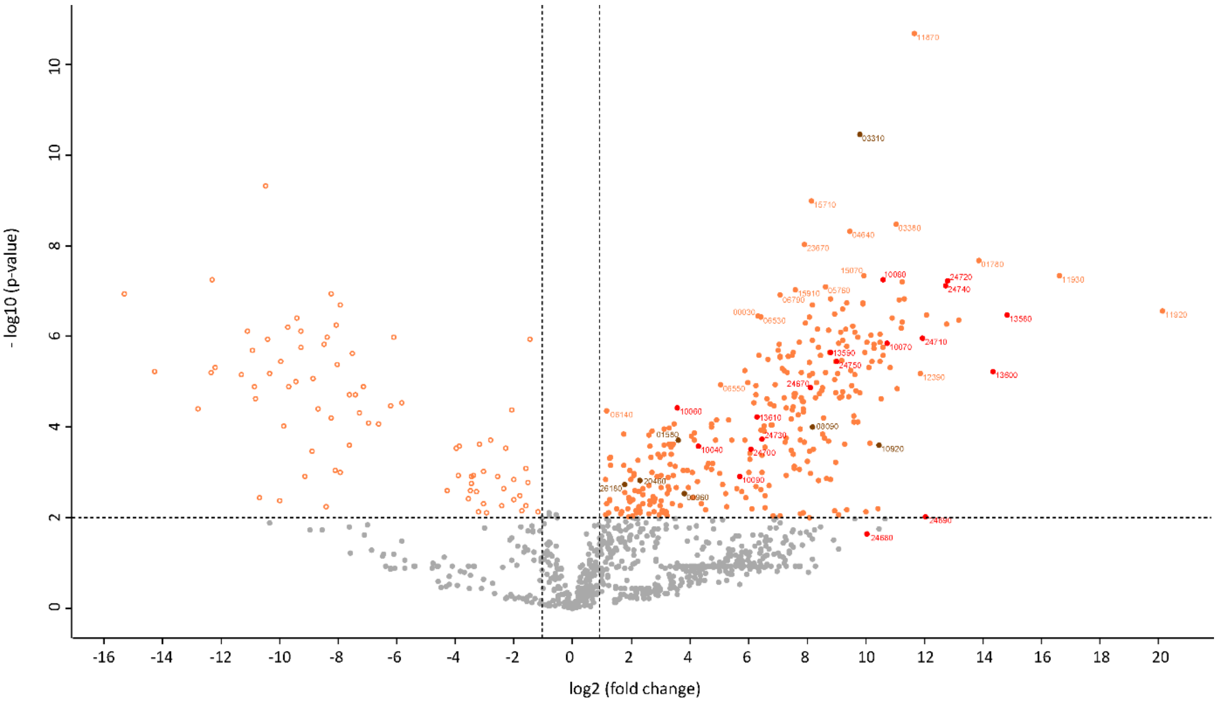

Supplementary Figure S7A. ‘Volcano plot’ of statistical significance (t-test analysis) against fold change of *Formosa* B protein abundances during growth on laminarin vs. growth on peptone. Filled orange dots with a p-value (-log10) >2 and a log2-fold change >1 represent laminarin up-regulated proteins. Open orange dots represent laminarin down-regulated proteins. Filled red dots display proteins of the three laminarin PULs, brown dots display upregulated peptidases or the highly expressed porin.

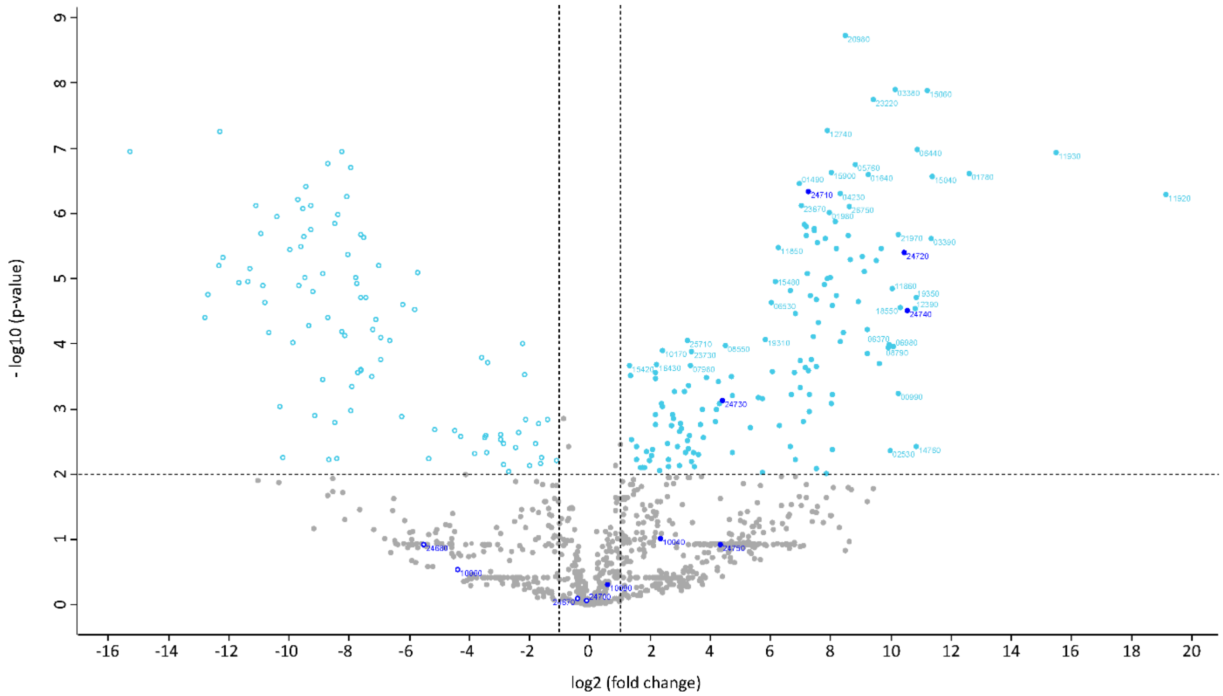

Supplementary Figure S7B. 'Volcano plot' of statistical significance (t-test analysis) against fold change of *Formosa* B protein abundances during growth on glucose vs. growth on peptone. Filled bright blue dots with a p-value (-log10) >2 and a log2-fold change >1 represent laminarin up-regulated proteins. Open bright blue dots represent glucose down-regulated proteins. Filled dark blue dots display identified proteins of three laminarin PULs.

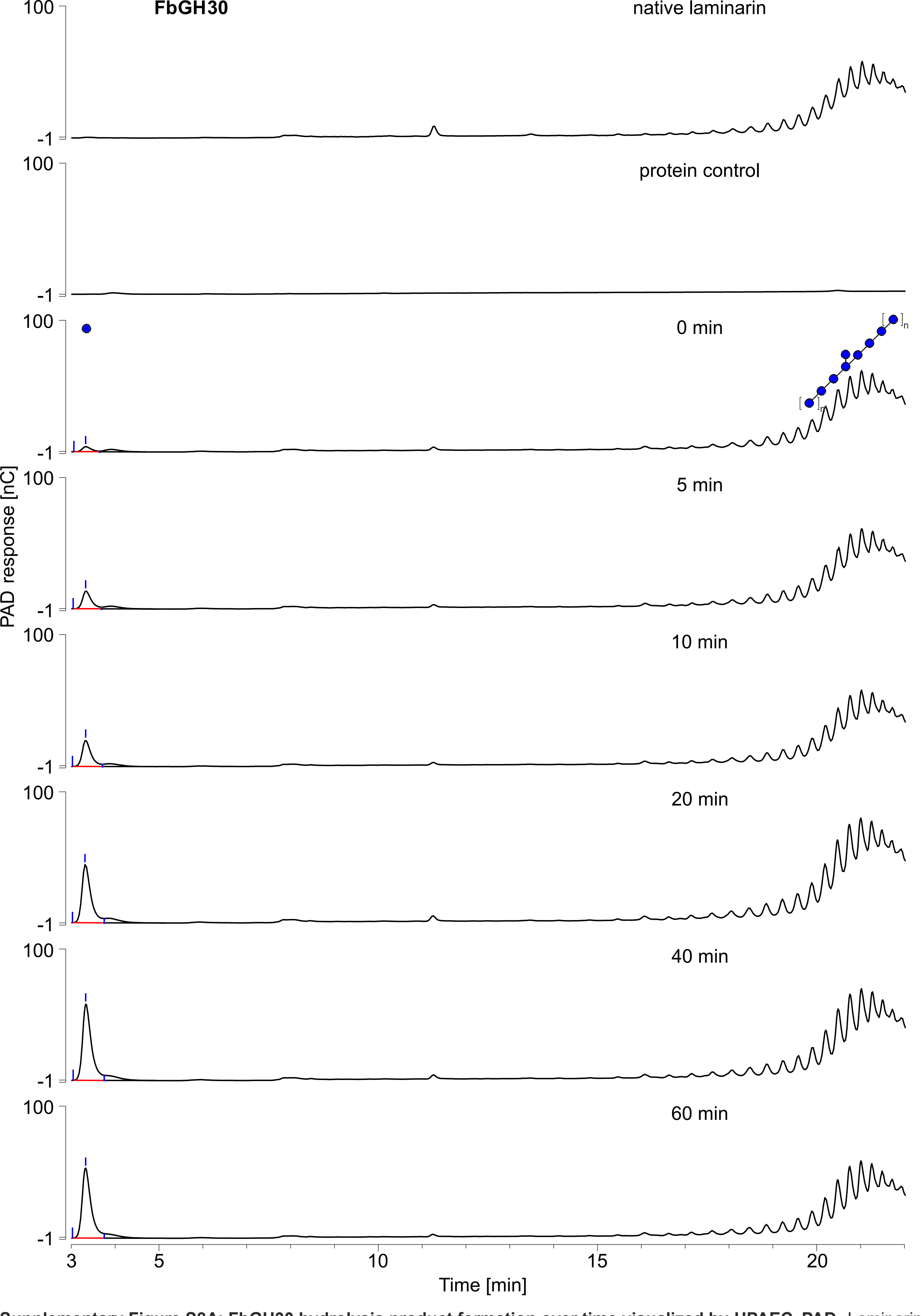

Supplementary Figure S8A: FbGH30 hydrolysis product formation over time visualized by HPAEC-PAD. Laminarin was hydrolyzed by the enzyme for 60 min (37°C, 50 mM MOPS buffer pH 7). Aliquots were taken at distinct time points and the reaction was stopped. FbGH30 was hydrolyzing native laminarin. The protein control contained only the enzyme without any substrate. After this debranching reaction, the laminarin was purified to remove glucose for the following steps. This debranched laminarin was used in the FbGH17A reaction. FbGH17B hydrolyzed the products of the previous FbGH17A reaction without any further purification in between.

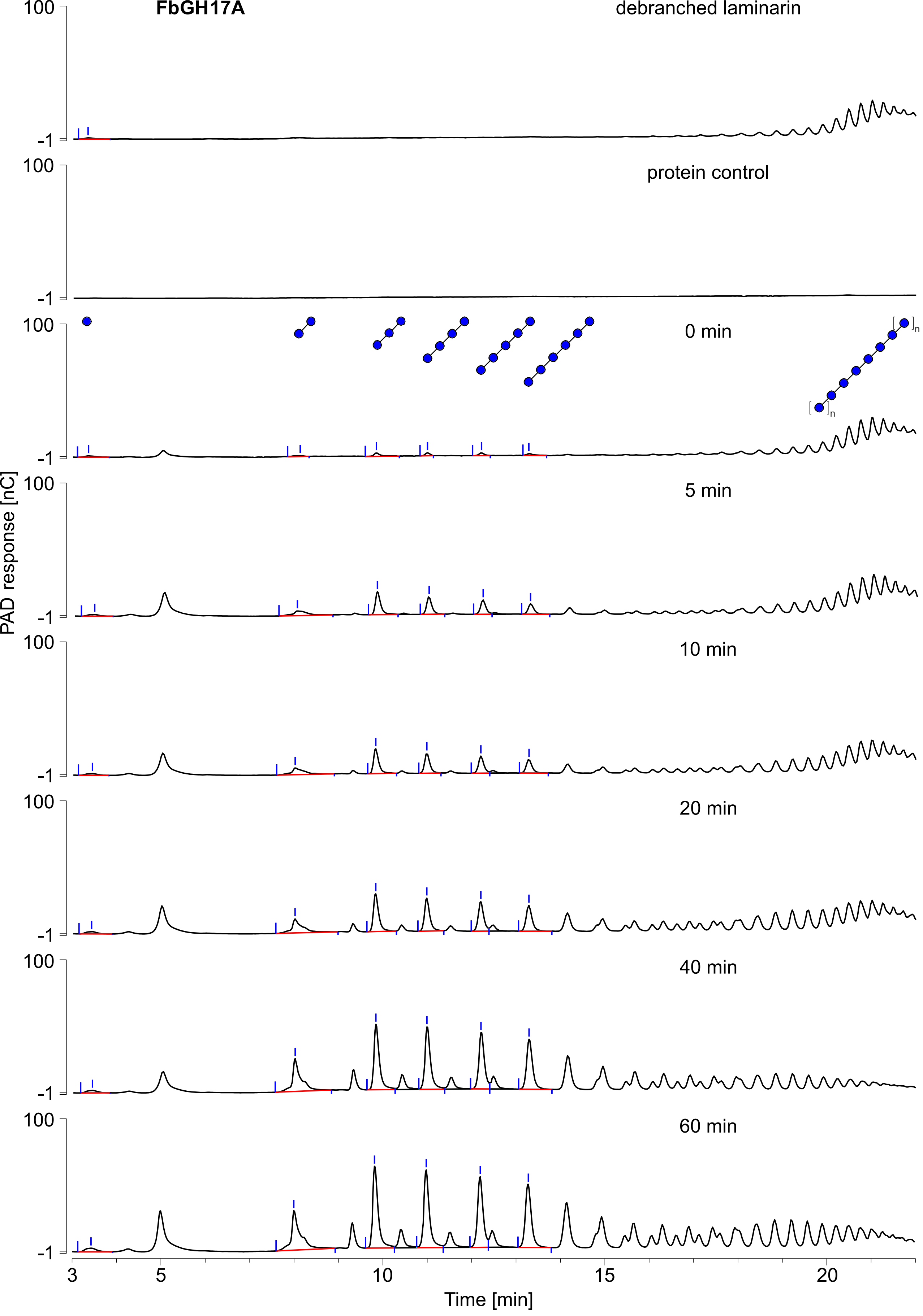

Supplementary Figure S8B: FbGH17A hydrolysis product formation over time visualized by HPAEC-PAD. Laminarin was hydrolyzed by the enzyme for 60 min (37°C, 50 mM MOPS buffer pH 7). Aliquots were taken at distinct time points and the reaction was stopped. FbGH17A was hydrolyzing debranched laminarin, which was purified from the previous FbGH30 digestion. The protein control contained only the enzyme without any substrate.

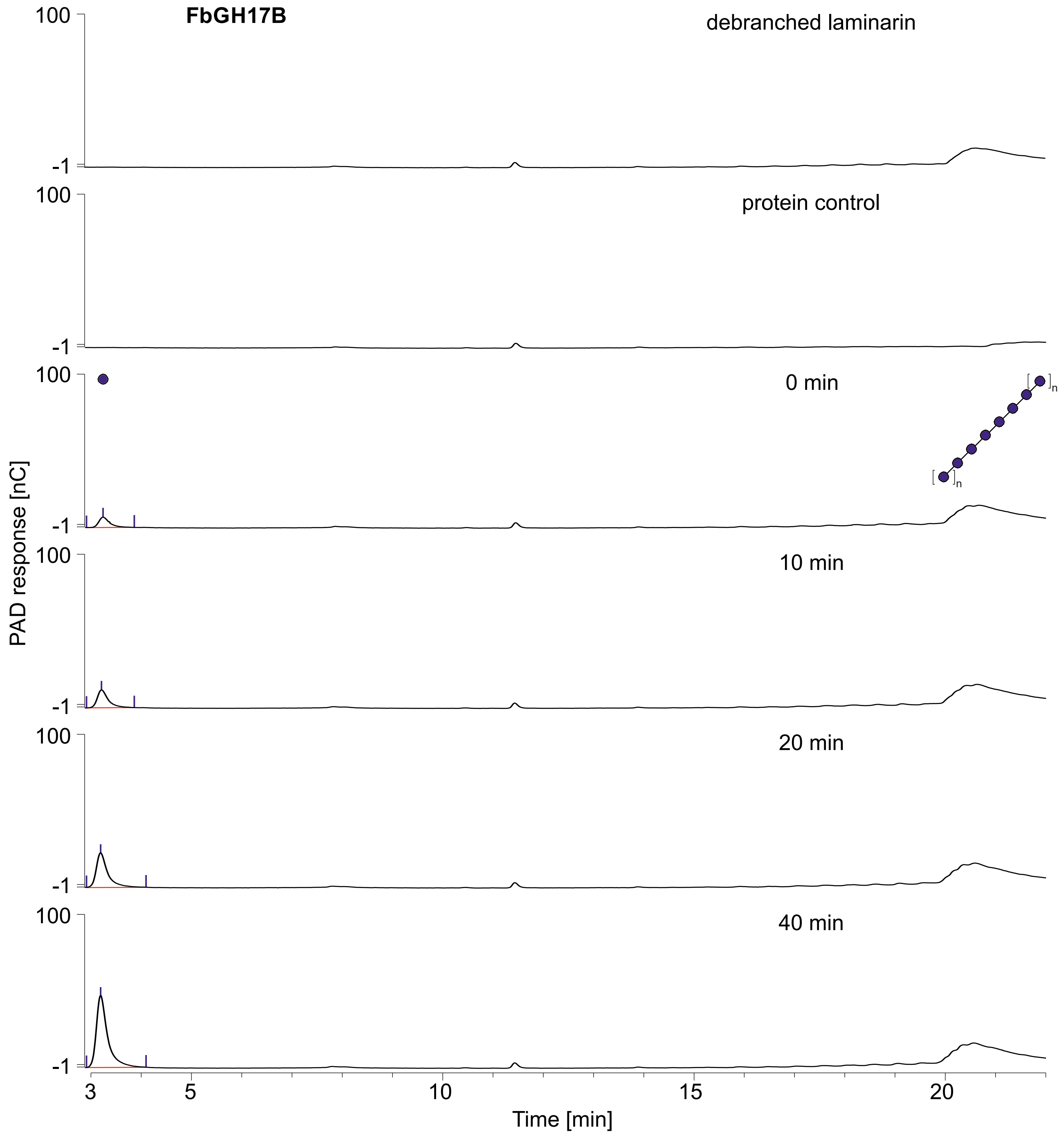

Supplementary Figure S8C: FbGH17B hydrolysis product formation over time visualized by HPAEC-PAD. Laminarin was hydrolyzed by the enzyme for 60 min (37°C, 50 mM MOPS buffer pH 7). Aliquots were taken at distinct time points and the reaction was stopped. FbGH17B was hydrolyzing debranched laminarin, which were derived from the previous FbGH30 digestion. The protein control contained only the enzyme without any substrate.

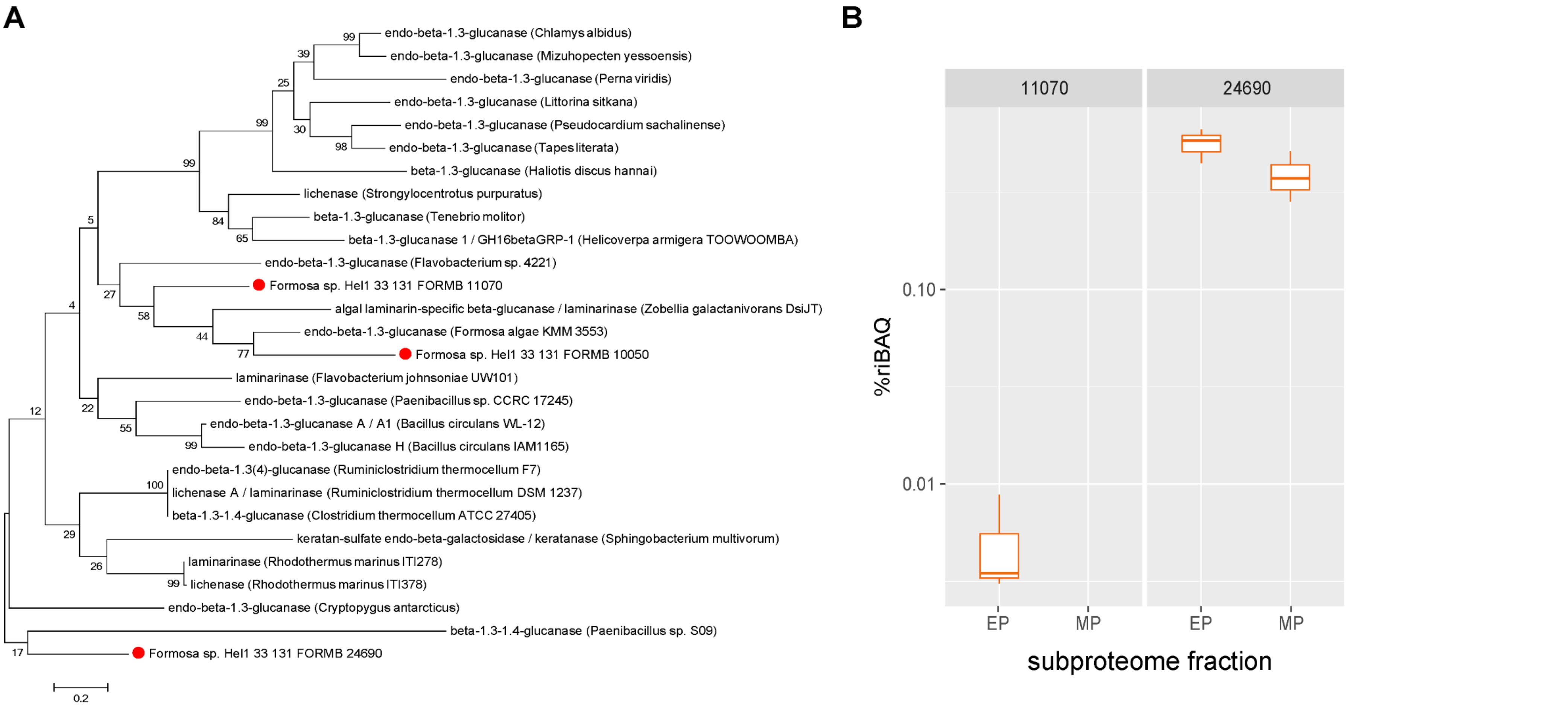

Supplementary Figure S9. GH16 enzymes in *Formosa* B. (**A**) Maximum likelihood phylogenetic tree of the three GH16 enzymes FORMB_11070, FORMB_24690 and FORMB_10050. The alignment was prepared with MUSCLE (Edgar, 2004) and the tree with MEGA6 (Tamura *et al.*, 2013). Numbers given on the branches are bootstrap proportions as a percentage of 1000 replicates. The scale bar represents the number of amino acid substitutions per site. (**B**) Abundance of the putative *Formosa* B laminarinases GH16 FORMB_11070 and FORMB_24690 in the presence of laminarin. None of these proteins could be detected during growth on glucose. The third GH16, FORMB_10050, was not at all detected in our *Formosa* B proteome analysis under either of the conditions. EP: enriched extracellular proteome, MP: membrane-associated proteome.

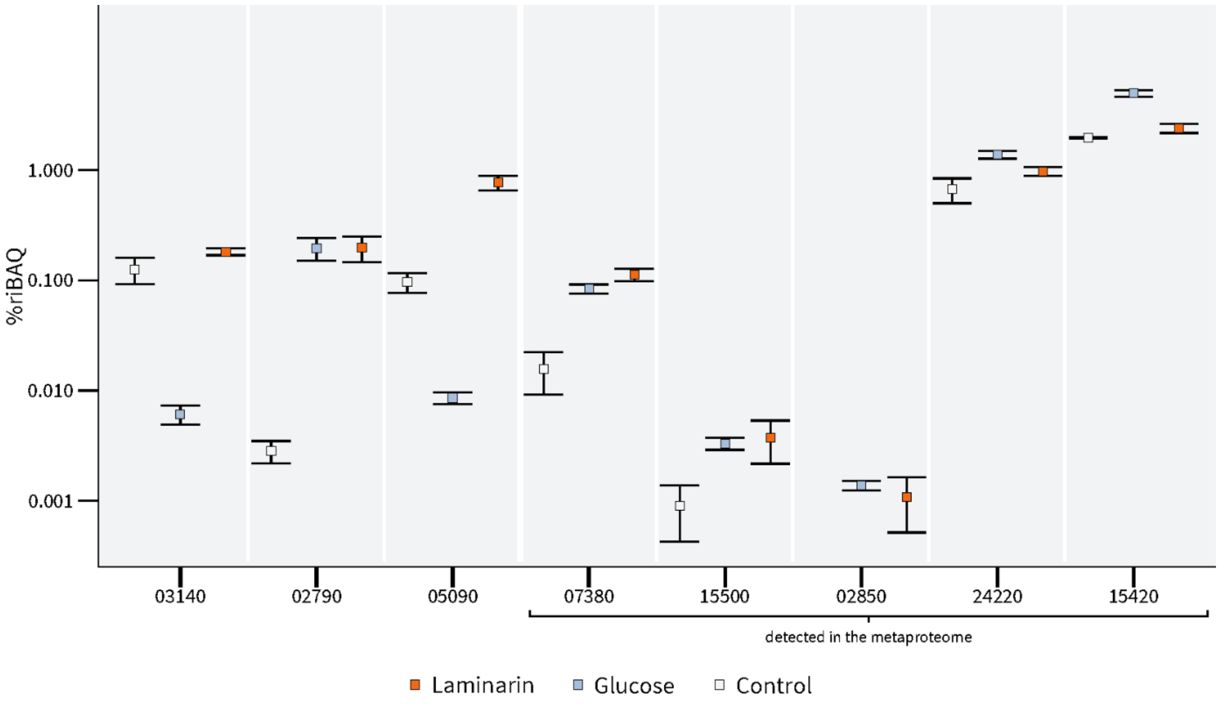

Supplementary Figure S10. Abundance of non-PUL-encoded TBDRs the expression of which were laminarin- and/or glucose-induced (see also Supplementary Tables S2A-C and S3). These proteins were detected in the membrane-enriched proteome of *Formosa* B. The five proteins shown on the right were also detected in the metaproteome of a spring bloom in 2009 (see Supplementary Table S8A). Relative protein abundances are depicted as %riBAQ values, i.e., as % of all proteins in the same sample. The respective locus tags of the proteins are indicated. The squares represent the mean values of the three replicates for every protein and each substrate. The error bars refer to the standard error of the mean. Proteins that could be detected in at least 2 out of 3 independent biological replicates of the investigated substrate conditions are shown (for individual replicate numbers see Supplementary Table S2A).

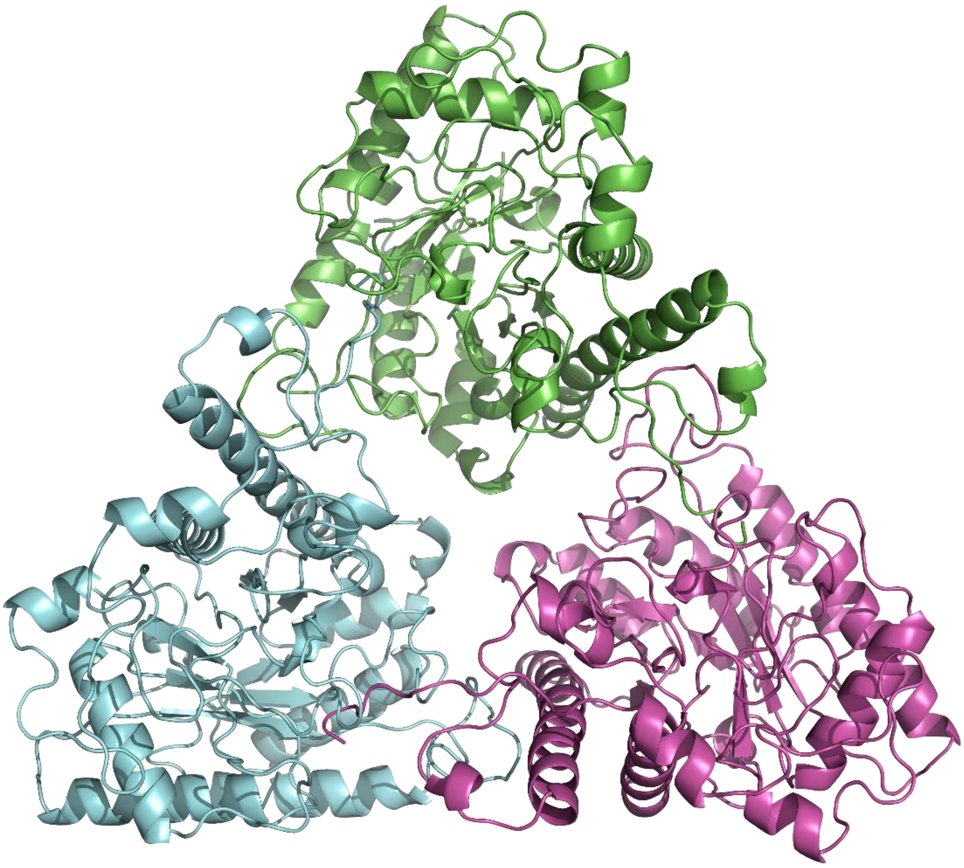

Supplementary Figure S11. Trimer of FbGH17A found in the asymmetric unit. The assembly is shown with each of the three monomers in cyan, green and purple.

References

Antelmann H, Tjalsma H, Voigt B, Ohlmeier S, Bron S, van Dijl JM *et al* (2001). A proteomic view on genome-based signal peptide predictions. *Genome Res* **11:** 1484-1502.

Arnosti C (2003). Fluorescent derivatization of polysaccharides and carbohydrate-containing biopolymers for measurement of enzyme activities in complex media. *J Chromatogr B Analyt Technol Biomed Life Sci* **793:** 181-191.

Berven FS, Flikka K, Jensen HB, Eidhammer I (2004). BOMP: a program to predict integral beta-barrel outer membrane proteins encoded within genomes of Gram-negative bacteria. *Nucleic Acids Res* **32:** W394-399.

Bushnell B (2016). BBMap. *sourceforgenet/projects/bbmap/*.

Camacho C, Coulouris G, Avagyan V, Ma N, Papadopoulos J, Bealer K *et al* (2009). BLAST+: architecture and applications. *BMC Bioinformatics* **10:** 421.

Candiano G, Bruschi M, Musante L, Santucci L, Ghiggeri GM, Carnemolla B *et al* (2004). Blue silver: a very sensitive colloidal Coomassie G-250 staining for proteome analysis. *Electrophoresis* **25:** 1327-1333.

Edgar RC (2004). MUSCLE: multiple sequence alignment with high accuracy and high throughput. *Nucleic Acids Res* **32:** 1792-1797.

Gibson DG, Young L, Chuang RY, Venter JC, Hutchison CA, 3rd, Smith HO (2009). Enzymatic assembly of DNA molecules up to several hundred kilobases. *Nat Methods* **6:** 343-345.

Guy L, Kultima JR, Andersson SG (2010). genoPlotR: comparative gene and genome visualization in R. *Bioinformatics* **26:** 2334-2335.

Hahnke RL, Bennke CM, Fuchs BM, Mann AJ, Rhiel E, Teeling H *et al* (2015). Dilution cultivation of marine heterotrophic bacteria abundant after a spring phytoplankton bloom in the North Sea. *Environ Microbiol* **17:** 3515-3526.

Hehemann JH, Kelly AG, Pudlo NA, Martens EC, Boraston AB (2012). Bacteria of the human gut microbiome catabolize red seaweed glycans with carbohydrate-active enzyme updates from extrinsic microbes. *Proceedings of the National Academy of Sciences of the United States of America* **109:** 19786-19791.

Heinz E, Williams TA, Nakjang S, Noel CJ, Swan DC, Goldberg AV *et al* (2012). The genome of the obligate intracellular parasite Trachipleistophora hominis: new insights into microsporidian genome dynamics and reductive evolution. *PLoS Pathog* **8:** e1002979.

Jones DT, Taylor WR, Thornton JM (1992). The rapid generation of mutation data matrices from protein sequences. *Comput Appl Biosci* **8:** 275-282.

Kabisch A, Otto A, Konig S, Becher D, Albrecht D, Schuler M *et al* (2014). Functional characterization of polysaccharide utilization loci in the marine Bacteroidetes 'Gramella forsetii' KT0803. *ISME J* **8:** 1492-1502.

Li H, Handsaker B, Wysoker A, Fennell T, Ruan J, Homer N *et al* (2009). The Sequence Alignment/Map format and SAMtools. *Bioinformatics* **25:** 2078-2079.

Ludwig W, Strunk O, Westram R, Richter L, Meier H, Yadhukumar *et al* (2004). ARB: a software environment for sequence data. *Nucleic Acids Res* **32:** 1363-1371.

Moretti R, Thorson JS (2008). A comparison of sugar indicators enables a universal high-throughput sugar-1-phosphate nucleotidyltransferase assay. *Anal Biochem* **377:** 251-258.

Peplies J, Kottmann R, Ludwig W, Glockner FO (2008). A standard operating procedure for phylogenetic inference (SOPPI) using (rRNA) marker genes. *Syst Appl Microbiol* **31:** 251-257.

Pruesse E, Peplies J, Glockner FO (2012). SINA: accurate high-throughput multiple sequence alignment of ribosomal RNA genes. *Bioinformatics* **28:** 1823-1829.

Qi X, Sun Y, Xiong S (2015). A single freeze-thawing cycle for highly efficient solubilization of inclusion body proteins and its refolding into bioactive form. *Microb Cell Fact* **14:** 24.

Qin Z, Yan Q, Lei J, Yang S, Jiang Z, Wu S (2015). The first crystal structure of a glycoside hydrolase family 17 beta-1,3-glucanosyltransferase displays a unique catalytic cleft. *Acta Crystallogr D Biol Crystallogr* **71:** 1714-1724.

Quast C, Pruesse E, Yilmaz P, Gerken J, Schweer T, Yarza P *et al* (2013). The SILVA ribosomal RNA gene database project: improved data processing and web-based tools. *Nucleic Acids Res* **41:** D590-596.

Reintjes G, Arnosti C, Fuchs BM, Amann R (2017). An alternative polysaccharide uptake mechanism of marine bacteria. *ISME J* **11:** 1640-1650.

Romine MF (2011). Genome-wide protein localization prediction strategies for gram negative bacteria. *BMC Genomics* **12 Suppl 1:** S1.

Sato K, Naito M, Yukitake H, Hirakawa H, Shoji M, McBride MJ *et al* (2010). A protein secretion system linked to bacteroidete gliding motility and pathogenesis. *Proceedings of the National Academy of Sciences of the United States of America* **107:** 276-281.

Schneider T, Schmid E, de Castro JV, Jr., Cardinale M, Eberl L, Grube M *et al* (2011). Structure and function of the symbiosis partners of the lung lichen (Lobaria pulmonaria L. Hoffm.) analyzed by metaproteomics. *Proteomics* **11:** 2752-2756.

Stamatakis A (2014). RAxML version 8: a tool for phylogenetic analysis and post-analysis of large phylogenies. *Bioinformatics* **30:** 1312-1313.

Studier FW (2005). Protein production by auto-induction in high-density shaking cultures. *Protein expression and purification* **41:** 207-234.

Tamura K, Stecher G, Peterson D, Filipski A, Kumar S (2013). MEGA6: Molecular Evolutionary Genetics Analysis version 6.0. *Mol Biol Evol* **30:** 2725-2729.

Teeling H, Fuchs BM, Becher D, Klockow C, Gardebrecht A, Bennke CM *et al* (2012). Substrate-controlled succession of marine bacterioplankton populations induced by a phytoplankton bloom. *Science* **336:** 608-611.

Teeling H, Fuchs BM, Bennke CM, Kruger K, Chafee M, Kappelmann L *et al* (2016). Recurring patterns in bacterioplankton dynamics during coastal spring algae blooms. *Elife* **5:** e11888.

Thomas F, Lundqvist LC, Jam M, Jeudy A, Barbeyron T, Sandström C *et al* (2013). Comparative characterization of two marine alginate lyases from Zobellia galactanivorans reveals distinct modes of action and exquisite adaptation to their natural substrate. *Journal of Biological Chemistry* **288:** 23021-23037.

Varghese JN, Garrett T, Colman PM, Chen L, Høj P, Fincher GB (1994). Three-dimensional structures of two plant beta-glucan endohydrolases with distinct substrate specificities. *Proceedings of the National Academy of Sciences* **91:** 2785-2789.
